# Unsupervised segmentation of low-contrast multichannel images: discrimination of tissue components in microscopic images of unstained specimens

**DOI:** 10.1038/srep11576

**Published:** 2015-06-23

**Authors:** Ivica Kopriva, Marijana Popović Hadžija, Mirko Hadžija, Gorana Aralica

**Affiliations:** 1 Division of Laser and Atomic Research and Development, Ruđer Bošković Institute, Bijenička cesta 54, 10002 Zagreb, Croatia; 2 Division of Molecular Medicine, Ruđer Bošković Institute, Bijenička cesta 54, 10002 Zagreb, Croatia; 3 Department of Pathology and Cytology, Clinical Hospital Dubrava, Avenija Gojka Šuška 6, 10000 Zagreb, Croatia; 4 School of Medicine, University of Zagreb, Šalata 3, 10000 Zagreb, Croatia

## Abstract

Low-contrast images, such as color microscopic images of unstained histological specimens, are composed of objects with highly correlated spectral profiles. Such images are very hard to segment. Here, we present a method that nonlinearly maps low-contrast color image into an image with an increased number of non-physical channels and a decreased correlation between spectral profiles. The method is a proof-of-concept validated on the unsupervised segmentation of color images of unstained specimens, in which case the tissue components appear colorless when viewed under the light microscope. Specimens of human hepatocellular carcinoma, human liver with metastasis from colon and gastric cancer and mouse fatty liver were used for validation. The average correlation between the spectral profiles of the tissue components was greater than 0.9985, and the worst case correlation was greater than 0.9997. The proposed method can potentially be applied to the segmentation of low-contrast multichannel images with high spatial resolution that arise in other imaging modalities.

Segmentation of nontrivial images is considered one of the most difficult tasks in image processing^1^. Image segmentation refers to the partitioning of an image into sets of pixels (segments) corresponding to distinct objects^2^. Within the scope of this study, distinct objects refer to spectrally distinct tissue components present in the images of unstained specimens. It is important to distinguish between single (grayscale)- and multi-channel images. In the former case, segmentation is performed by detection of changes of intensity or texture by thresholding some type of spatial derivative of an image^3^,^4^,^5^,^6^,^7^. However, images that comprise components with very similar profiles (spectral, density, and/or concentration) have very low visual contrast. For an example, if staining is not used, the spectral similarity between the tissue components present in the specimen is very high and the visual contrast is very poor, i.e., tissue components appear colorless and virtually texture-less when viewed under a light microscope. This situation occurs in the case of synthetic images (Fig. 1a), as well as in case of color microscopic images of unstained specimens of human hepatocellular carcinoma (primary liver tumor) (Fig. 2a), liver tissue with metastasis from colon cancer (Fig. 3a), and gastric cancer (Fig. 4a). Thus, when spectral vectors are plotted vs. their indices (corresponding red, green and blue colors) they are virtually parallel (Figs. 1g,2g and Figs 2a,3a,4a, S5a and S6a). Consequently, the intensity and/or texture-based segmentation methods^3^,^4^,^5^,^6^,^7^ fail to segment tissue components correctly (Figures S2b and S2c). Segmentation of the color image by means of clustering in the CIE L^*^a^*^b^*^ color space^8^ also fails for the same reason (Fig. 1e, S2a, S3a, S4a, S5e and S6f).

Multichannel images, such as color microscopic images, can also be segmented by means of multivariate data analysis methods, such as independent component analysis^9^, nonnegative matrix factorization (NMF)^10^ and sparse component analysis^11^. These methods are known under the common name of blind source separation^12^. They yield analog values rather than binary values and can be interpreted as the probability that a pixel belongs to a specific object. The segmented image binary outcome is obtained by thresholding the analog values of the decomposed encoding coefficients (i.e., sources). Blind source separation methods decompose color images into constituent components through factorization. To this end, the unfolded color image **X** is represented by a linear mixture model (equation (1)) (Supplementary Material 1):


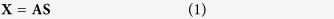


Thereby, 
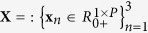
 comprises vectorized intensity images, each *P* pixel elements in size, corresponding to red, green and blue colors. The symbol =: means “by definition”. Without loss of generality, it is assumed that 
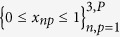
, i.e., the intensity images are scaled to [0, 1]. 
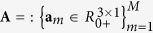
 stands for the basis matrix such that the basis vectors represent spectral profiles of the *M* tissue components present in image **X**. 
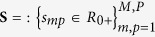
 stands for the source matrix with the coefficients *s*_*mp*_ that encode the amount of presence of a tissue component *m* at pixel *p*. Constraints such as sparseness and non-negativity have to be imposed on **S** and **A** to ensure the decomposition (1) is unique with scaling and permutation ambiguities only (Supplementary Material 1). Unique decomposition is necessary to distinguish between healthy and diseased tissues. For this reason, we impose binary and orthogonality constraints on the coefficients of matrix **S**: 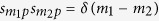, where *δ* stands for the Kronecker delta. The binary constraint is the natural choice for image segmentation problems. It indicates whether tissue component *m* is present, 1, or absent, 0, at pixel *p*. The orthogonality constraint implies that at each pixel, only one tissue component is present. This constraint is justified when the spatial resolution of the light microscope is high enough. The binary constraint has been used in the separation of sources from linear^13^,^14^ and nonlinear^15^,^16^ mixtures. Separation is possible when the spectral profiles that correspond to mixing vectors, 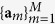, are distinctive enough. However, low contrast images contain tissue components 
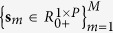
 with highly correlated spectral profiles. Hence, it is virtually impossible to decompose (segment) these images by the state-of-the-art methods.

This study demonstrates that the separation of tissue components with highly correlated spectral profiles, such as those present in the color microscopic images of unstained specimens, is possible (Figs 2c,3c,4c as well as Figures S5d, S6d and S6e). To this end, a method that performs sparseness-constrained NMF in a reproducible kernel Hilbert space (RKHS) is proposed (Methods and Supplementary Material 1 to 4).

## Results

### Sparse nonnegative matrix factorization in RKHS

#### Empirical kernel map

**Figure 1:**
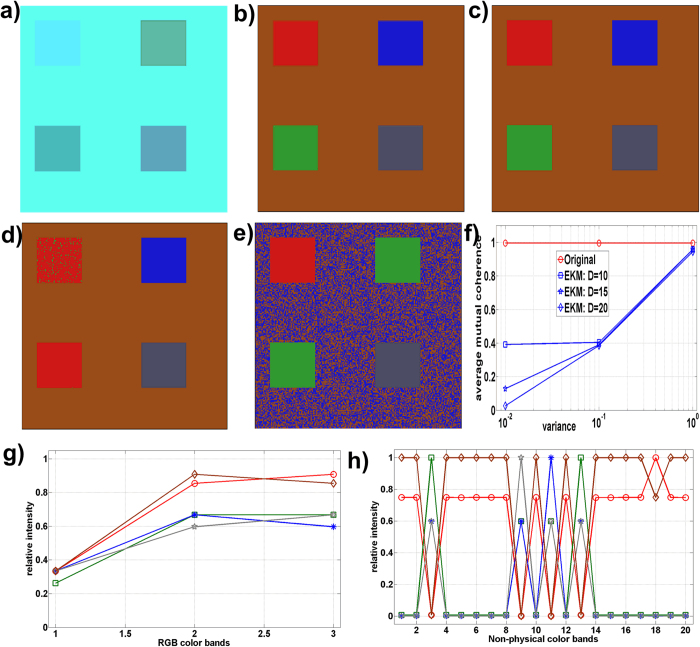
(**a**) Synthetic image in red-green-blue color space composed of five spectrally highly similar objects. There is additive white Gaussian noise in each spectral channel with SNR = 70 dB. (**b**) color-coded ground truth image. (**c**) color-coded image of the segmentation result obtained by the EKM-NMU algorithm (Gaussian kernel with σ^2^ = 0.01 and D = 20). (**d**) color-coded image of the segmentation result obtained by the NMU algorithm. (**e**) color-coded image of the segmentation result obtained by the k_means algorithm in the CIE L^*^a^*^b^*^ color space. (**f**) μ_average(**A**) for basis matrix **A** in (1) and μ_average(**B**) for basis matrix **B** in the EKM-induced space (2). (**g**) spectral responses of five objects in red (1)-green(2)-blue(3) color space, μ(**A)**=0.9995, μ_average(**A**) = 0.9956. (**h**) spectral responses of five objects in non-physical color space induced by EKM (D=20, Gaussian kernel with σ^2^ = 0.01), μ(**B**) = 0.9807, μ_average(**B**) = 0.3777.

**Figure 2:**
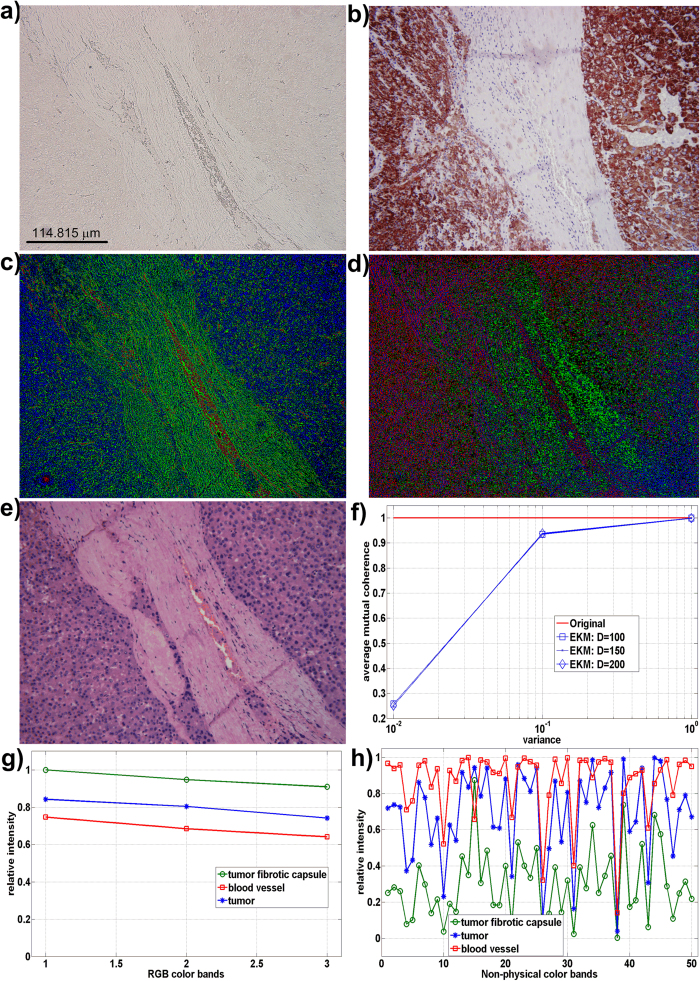
Human liver with hepatocellular carcinoma. (**a**) red-green-blue (RGB) color microscopic image of unstained specimen. (**b**) “ground truth – different slides” RGB color microscopic image of the specimen stained by Hep Par. (**c**) color-coded (digitally stained) image of the segmentation result obtained by the EKM-NMF_L0 algorithm (Gaussian kernel with variance = 0.1 and D = 50): blue: hepatocellular carcinoma, red: blood vessel, green: tumor fibrotic capsule. (**d**) color-coded (digitally stained) image of the segmentation result obtained by the NMF_L0 algorithm. (**e**) RGB color microscopic image of the specimen (a) stained subsequently by H&E. (**f**) average mutual coherence for the original matrix of the spectral profiles and matrices in the EKM-induced space. (**g**) spectral responses of the tumor fibrotic capsule, blood vessel and hepatocellular carcinoma in RGB (1-2-3) color space, μ(**A**) > 0.9999, μ_average(**A**) = 0.9985. (**h**) spectral responses of the tumor fibrotic capsule, blood vessel and hepatocellular carcinoma in non-physical color space induced by EKM (D = 50, Gaussian kernel variance=0.1), μ(**B**) = 0.9760. μ_average(**B**) = 0.8937.

Here, we propose nonlinear mapping of (1) to obtain (Supplementary Material 3):


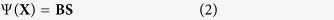


where 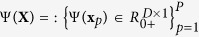, 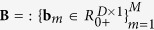 and 3 < *D ≤ P*. *D* stands for the dimension of a subspace in the infinite dimensional RKHS onto which the original image **X** is mapped. The mapping Ψ(**X**) is known as an empirical kernel map (EKM), see definition 2.15 in ref.^17^(Supplementary Material 3). The EKM-based mapping Ψ(**X**), in combination with nonnegativity and sparseness constraints, has been used to separate analytes from nonlinear mixtures in mass spectra^18^. However, binary {0, 1} and orthogonality constraints are imposed on 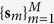 herein for the first time. It is a necessary condition for Ψ(**X**) to be invariant with respect to the sources 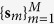 (Supplementary Material 3). The parameters of the EKM (subspace dimension *D* and variance σ^2^ of the Gaussian kernel) are optimized to decrease the correlation between basis vectors 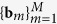 in (2) in comparison with the correlation between basis vectors 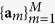 in (1) (Figs 1f,h,2f,h) (Supplementary Material 3). This enables segmentation of the tissue components in the color microscopic images of unstained specimens. However, the characteristics of the linear mixture model (2) are also important for segmentation of low-contrast multichannel images with high spatial resolution that arise in other imaging modalities.

#### Sparseness-constrained NMF

After EKM-based mapping of Ψ(**X**), the basis vectors 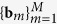 in (2) are less correlated than the basis vectors 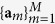 in (1), decomposition of the image Ψ(**X**) in (2) under binary and orthogonality constraints on 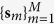 will more probably yield meaningful, i.e., interpretable, results than the decomposition of **X** in (1). In particular, stated constraints are built into the NMF method^19^. They were implemented through minimization of the number of nonzero coefficients of the column vectors of matrix **S** in (1)/(2) (the NMF_L0 method, where L0 stands for quasi norm that counts the number of nonzero coefficients). We named the method that combines the EKM mapping of Ψ(**X**) and the NMF_L0 factorization of Ψ(**X**) the EKM-NMF_L0 method. Alternatively, it is possible to use the nonnegative matrix underapproximation (NMU) algorithm^20^ to indirectly ensure sparse decomposition of (1)/(2). We named the method that combines the EKM mapping of Ψ(**X**) and the NMU factorization of Ψ(**X**) as the EKM-NMU method.

#### Intrinsic dimension or model order selection

The number of tissue components *M* present in linear mixture models (1) and (2) is known as the model order or intrinsic dimension. Its determination is a challenging problem in multivariate data analysis^21^. Several methods for model order selection have been developed with various applications within signal processing and data analysis^22^. However, no model order selection method works satisfactorily when used alone^23^. We therefore rely on the *a priori* knowledge (experience) that the number of tissue components *M* present in histopathological specimens is approximately 4 to 6. Thus, when the factorization of the linear mixture models (1) and/or (2) was performed, *M* was set to the specified predefined value.

### Segmentation of synthetic low-contrast color images

**Figure 3:**
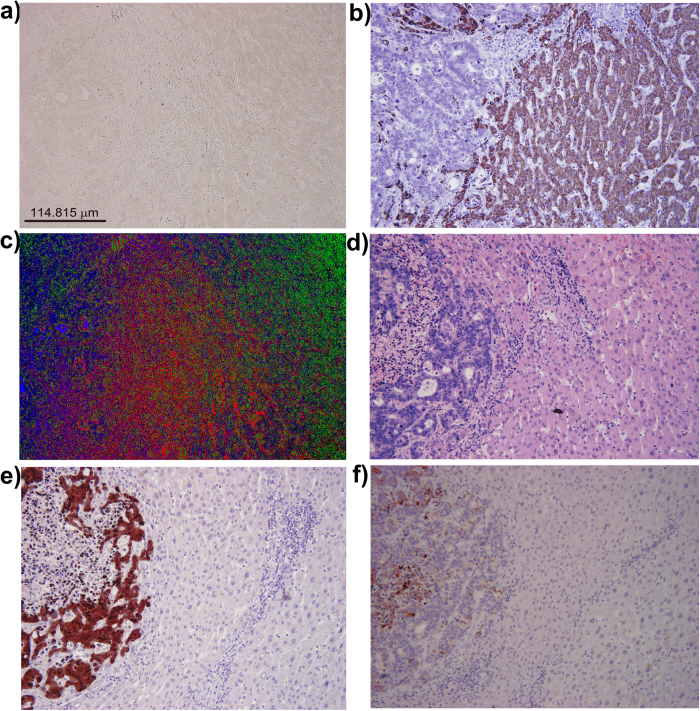
Human liver with metastasis of colon cancer. (**a**) red-green-blue (RGB) color microscopic image of unstained specimen. μ(**A**) = 0.9997, μ_average(**A**) = 0.9993. (**b**) “ground truth – subsequent slides” RGB color microscopic image of the specimen stained by Hep Par. (**c**) color-coded (digitally stained) image of the segmentation result obtained by the EKM-NMF_L0 algorithm (Gaussian kernel variance = 0.1 and D = 50): blue: metastasis of colon cancer, red: border area between tumor and liver tissue, green: hepatocytes. μ(**B**) = 0.9998, μ_average(**B**) = 0.9984. (**d**) RGB color microscopic image of the specimen (**a**) stained subsequently by H&E. (**e**) “ground truth – subsequent slides” RGB color microscopic image of the specimen stained by CDX2. (**f**) “ground truth - subsequent slides” RGB color microscopic image of the specimen stained by CK20.

**Figure 4:**
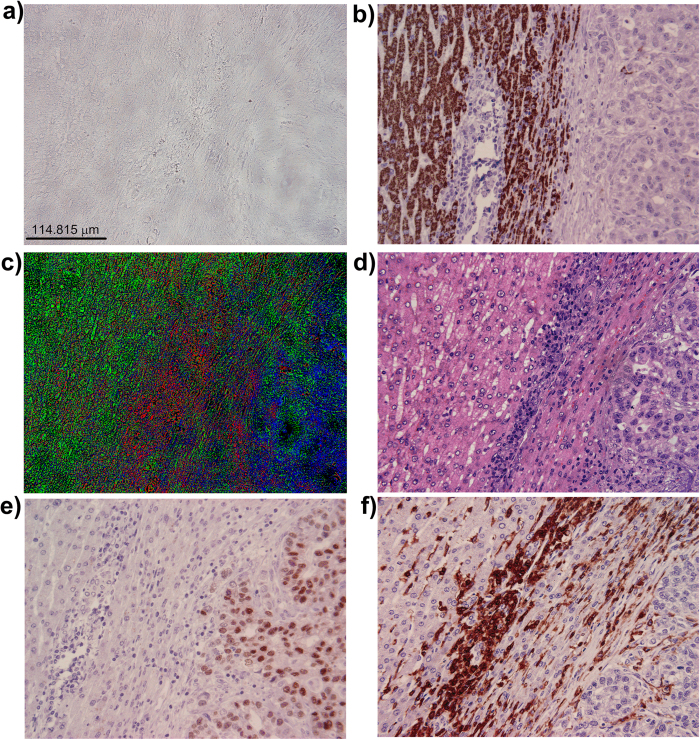
Human liver with metastasis of gastric cancer. (**a**) red-green-blue (RGB) color microscopic image of unstained specimen. μ(**A**) = 0.9999, μ_average(**A**) = 0.9988. (**b**) “ground truth – subsequent slides” RGB color microscopic image of the specimen stained by Hep Par. (**c**) color-coded (digitally stained) image of the segmentation result obtained by EKM-NMF_L0 algorithm (Gaussian kernel variance = 0.1 and D = 50): blue: metastasis of gastric cancer, red: border area of inflammation, green: hepatocytes. μ(**B**) = 0.9994, μ_average(**B**) = 0.9917. (**d**) RGB color microscopic image of specimen (a) stained subsequently by H&E. (**e**) “ground truth – subsequent slides” RGB color microscopic image of the specimen stained by CDX2. (**f**) “ground truth - subsequent slides” RGB color microscopic image of the specimen stained by LCA.

To validate and quantify the performance of the proposed EKM-based approach to the segmentation of low-contrast multichannel images, an RGB color image was synthesized by computer (Fig. 1a). The image is composed of five spectrally highly similar objects. The color-coded ground truth image is given in Fig. 1b, which is also known as region coloring, i.e., assigning colors to the pixels such that different colors correspond to different objects, and is commonly used to display segmentation results. Region coloring yields a compact representation of an image in terms of its constituent parts. The image in Fig. 1a is generated according to a linear mixture model (1), with orthogonal and {0, 1} binary sources and a mixing matrix with the maximal normalized correlation between two basis vectors equal to μ(**A**) = 0.9995, which is also known as mutual coherence, and an average correlation between basis vectors equal to μ_average(**A**)=0.9956 (Fig. 1f,g) (Methods and Supplementary Material 2). The mixing matrix in (1) is given in Table 2. The correlation properties between the basis vectors in **A** resemble the correlation properties between the spectral profiles of tissue components present in the images of the unstained specimens in Figs 2a,3a,4a. In addition to the original model (1), additive white Gaussian noise has been added to each spectral channel with a signal-to-noise (SNR) ratio equal to SNR = 70 dB, i.e., the noise power was 10 million times less than the signal power. Due to the high spectral similarity of the objects present in the image, this addition was enough to influence the result of the k-means clustering algorithm (Fig. 1e) and cause the NMU algorithm to fail to accurately decompose the synthetic image (Fig. 1d).

#### Gain in spectral diversity due to the empirical kernel map

By optimizing the parameters of the EKM, it was possible to decrease the correlation among the basis vectors 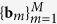 in (2) as opposed to the basis vectors 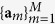 in (1) (Fig. 1f). This decrease correlation is clear when their values are plotted against the color channel indices (Fig. 1g) and against the indices of the non-physical channels in the mapped space (Fig. 1h), which explains the correct segmentation result obtained by means of the EKM-NMU algorithm (Fig. 1c). The reason for using the NMU algorithm instead of the NMF_L0 algorithm, which was used to decompose the images of the unstained specimens of human and mouse liver, is to demonstrate that the ability to separate spectrally highly similar objects is primarily a consequence of EKM-based mapping and secondarily of the sparseness-constrained NMF algorithm. After the EKM-based mapping of Ψ(**X**) parameterized with σ^2^ = 0.1 and *D *= 20, the μ(**A**) = 0.9995 and μ_average(**A**) = 0.9956 were, respectively, decreased to μ(**B**) = 0.9807 and μ_average(**B**) = 0.3777. In particular, the EKM-NMU method with σ^2^ = 0.1 and *D *= 20 yields correct segmentation of the synthetic image, with a per-channel SNR as low as 14 dB. Values of σ^2^ = 0.01 and σ^2^ = 0.001 decrease the mutual coherence even further, but it requires SNR values of at least 18 dB and 29 dB, respectively (Figure S1). SNR-based analysis (Supplementary Material 1 and Figure S1) enables parameterization of the EKM-NMU (NMF_L0) method as a function of the SNR value as follows. To separate histological structures with μ(**A**) ≤ 0.9998, set the dimensions of the EKM to *D *≥ 20 and the variance of the Gaussian kernel *σ*^2^ according to Table 1.

### Segmentation of color microscopic images of unstained specimens of liver

#### Construction of the ground truth image set

We applied the EKM-NMF_L0 algorithm to the segmentation of color light microscopic images of deparafinized unstained tissue sections of human hepatocellular carcinoma (Fig. 2a), human liver with metastasis from colon (Fig. 3a) and gastric (Fig. 4a) cancers, as well as to segmentation of the images of unstained cryosections of mouse fatty liver (Figures S5a and S6a). Evaluation of the performance of the segmentation algorithms is a nontrivial problem when experimental (clinical) images are to be segmented. In such a case, it is required that humans manually segment the set of training images to be used to quantify the algorithm performance^2^, which is a challenging task in images of the specimens used in histopathology. Quantitative performance analysis using a similarity metric, such as Dice’s coefficient, between segmented components and the ground truth-based gold standard requires manual segmentation of the images of unstained specimens by a pathologist, which is virtually impossible to perform with a decent accuracy. Thus, the gold standard is inaccurate, which is the reason that a qualitative type of performance analysis is performed herein. To this end, what is referred to as human segmentation is actually a set of color microscopic images of stained subsequent sections of the same specimen. Subsequent slides are stained to mark the targeted tissue components known by pathologists to be present in a specimen with a particular diagnosis. The slide is stained by hematoxylin and eosin (H&E), which is one of the principal stains used in histology, after the image of the unstained specimen has been acquired. Subsequent sections are then stained with other dyes suggested by the pathologist. The results obtained by the segmentation algorithms are then compared visually with this “ground truth” image set.

#### Segmentation of color microscopic image of unstained specimen of human hepatocellular carcinoma

The ground truth image set related to the image of the unstained specimen of human hepatocellular carcinoma (Fig. 2a) is obtained by immunohistochemical staining for hepatocyte antigen: Hepatocyte Clone OCH1E5 (Hep Par) (Fig. 2b) and H&E stain (Fig. 2e). Due to the absence of staining, the image of the unstained specimen appears colorless and almost texture-less. The segmentation result obtained by the EKM-NMF_L0 algorithm is shown in the color-coded image in Fig. 2c, where the hepatocellular carcinoma tissue is colored blue, blood vessel is colored red and the tumor fibrotic capsule is colored green. In the image of the Hep Par stained section (Fig. 2b), hepatocytes are colored brown, the endothelium of blood vessel is colored blue, and tumor fibrotic capsule is colored white. In the image of the H&E stained section (Fig. 2e), which is obtained by staining the specimen after the image shown in Fig. 2a is acquired, the hepatocytes, blood vessel and tumor fibrotic capsule are colored in blue and dark pink, pink-white and light pink, respectively. The visual correspondence between the EKM-NMF_L0 result (Fig. 2c) and the ground truth (Fig. 2b,e) is evident. In contrast, the NMF_L0 algorithm (Fig. 2d), k-means clustering in the CIE L^*^a^*^b^*^ color space (Figure S2a), geometric active contour method for gray scale images (Figure S2b) and vector (color) images (Figure S2c) yields results that correspond poorly or do not correspond at all to the ground truth. Very recently, the ORTSEG algorithm was derived for unsupervised segmentation of histopathology images, in particular, images stained by H&E^24^. Using the Matlab implementation of the ORTSEG algorithm provided by the authors, we have applied it to the segmentation of the image of unstained specimen (Fig. 2a) and to the segmentation of image of H&E stained specimen, as shown in Fig. 2e. The digitally stained image obtained by ORTSEG-based segmentation of the image of the H&E stained specimen (upper left corner in Figure S2e) for 3 tissue components that were assumed to be present in the specimen partially resembles the visual appearance of the EKM-NMF_L0 digitally stained image shown in Fig. 2c. The red colored component in Figure S2e corresponds to the blue colored tumor component in Fig. 2c and the brown colored hepatocytes component in Fig. 2b. The blue colored component in Figure S2e corresponds imprecisely to the red colored blood vessel component in Fig. 2c and the pink-white colored blood vessel component in Fig. 2e. The green colored component in Figure S2e corresponds imprecisely to the green colored tumor fibrotic capsule component in Fig. 2c, the red/light pink colored tumor fibrotic capsule component in Fig. 2e and the white colored tumor fibrotic capsule in Fig. 2b. The visual correspondence between the ground truth images shown in Fig. 2b,e and the digitally stained images obtained by the ORTSEG-based segmentation of the image of the unstained specimen (upper left corner in Figure S2d) is lost. In addition to the lost visual correspondence, the segmented texture regions in the ORTSEG-segmented image are spatially disjoint, whereas in the ground truth images (Fig. 2b,e) and the segmented result obtained by the EKM-NMF_L0 algorithm (Fig. 2c), they are entangled. The EKM-NMF_L0-based segmentation of the image of the unstained specimen of human hepatocellular carcinoma also demonstrated decreased similarity between the spectral profiles of the tissue components. For the image of the unstained specimen shown in Fig. 2a, the following applies: μ(**A**) > 0.9999 and μ_average(**A**) = 0.9985. The EKM-based mapping Ψ(**X**) in (2), parameterized with σ^2^ = 0.1 and *D*=50, yields μ(**B**) = 0.9760 and μ(**B**)_average = 0.8937 (Methods and Supplementary Material 2). The reported correlation values for synthetic and experimental images were obtained with σ^2^ = 0.1, but Figs 1f,2f suggest that it is possible to further decrease the correlation between basis vectors 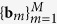
by decreasing σ^2^. However, the decrease depends on the signal-to-noise ratio because mapping Ψ(**X**) with too small σ^2^ makes it more sensitive to noise (Supplementary Material 3 and Table 1).

#### Segmentation of the color microscopic image of the unstained specimen of human liver with metastasis from colon cancer

The EKM-NMF_L0 algorithm was also applied to segment an image of the unstained specimen of human liver with metastasis from colon cancer (Fig. 3a). Due to the absence of staining, the image is colorless and almost texture-less. The ground truth images were obtained by staining the same section with H&E (Fig. 3d) and staining subsequent sections with Hep Par (Fig. 3b), CDX2 (Fig. 3e), and CK20 (Fig. 3f). In Hep Par, the ground truth image hepatocytes are colored brown, whereas the metastatic cells of colon cancer and the inflammatory cells are blue. In CDX2 and CK20, the ground truth images metastasis of colon cancer is colored completely or partially brown, whereas the hepatocytes and inflammatory cells are colored blue. The segmentation result of the EKM-NMF_L0 algorithm is shown in the color-coded image in Fig. 3c, where the metastasis of colon cancer is shown in blue, the border area between the tumor and liver tissue (fibrosis, inflammatory cells and a few hepatocytes) is in red and the hepatocytes are in green. The visual correspondence between the EKM-NMF_L0 result in Fig. 3c and the ground truth images in Fig. 3b,e,f is evident. In contrast, the segmentation results obtained by the NMF_L0 algorithm, shown in Figure S3b, the k-means clustering in the CIE L^*^a^*^b^*^ color space (Figure S3a), and the ORTSEG algorithm (Figures S3c and S3d), do not correspond visually to the ground truth images. EKM-NMF_L0-based segmentation of the image of the unstained specimen of human liver with metastasis from colon cancer also demonstrated decreased similarity between the spectral profiles of the tissue components. For the image of the unstained specimen shown in Fig. 3a, the following applies: μ(**A**) = 0.9997 and μ_average(**A**) = 0.9993. The EKM-based mapping of Ψ(**X**) in (2), parameterized with σ^2^ = 0.1 and *D *= 50, yields μ(**B**) = 0.9998 and μ(**B**)_average = 0.9984 (Methods and Supplementary Material 2). Although the average correlation decreased, the worst case correlation increased negligibly, which is a consequence of the coarse quantization of the intensity levels of the spectral images acquired with 8-bits per pixel. The image shown in Fig. 3a is demanding for segmentation.

#### Segmentation of the color microscopic image of the unstained specimen of human liver with metastasis of gastric cancer

The EKM-NMF_L0 algorithm was applied to the segmentation of an image of an unstained specimen of human liver with metastasis of gastric cancer (Fig. 4a). As before, due to the absence of staining, the image appears colorless and almost texture-less. The ground truth images were obtained by staining the same section with H&E (Fig. 4d) and by staining subsequent sections with Hep Par (Fig. 4b), CDX2 (Fig. 4e), and LCA (Fig. 4f). In Hep Par, the ground truth image hepatocytes are colored brown, and the metastatic cells from gastric cancer and the inflammatory cells are colored blue. In CDX2, the ground truth image metastasis of gastric cancer is colored brown, and the hepatocytes and inflammatory cells are colored blue. In LCA, the ground truth image inflammatory cells are colored brown and the hepatocytes and metastasis of gastric cancer are colored blue. The segmentation result of the EKM-NMF_L0 algorithm is shown in the color-coded image in Fig. 4c, where the metastasis of gastric cancer is shown in blue, the border area of the inflammation is shown in red and hepatocytes are shown in green. The visual correspondence between the EKM-NMF_L0 segmented image (Fig. 4c) and the ground truth images (Fig. 4b,e,f) is evident. In contrast, the segmentation results obtained by the NMF_L0 algorithm (Figure S4b), the k-means clustering in the CIE L^*^a^*^b^*^ color space (Figure S4a), and the ORTSEG algorithm (Figures S4c and S4d) do not correspond visually to the ground truth images. The EKM-NMF_L0-based segmentation of the image of the unstained specimen of human liver with metastasis of gastric cancer also demonstrated decreased similarity between the spectral profiles of the tissue components. For the image of the unstained specimen shown in Fig. 4a, the following applies: μ(**A**) = 0.9999 and μ_average(**A**) = 0.9988. The EKM-based mapping of Ψ(**X**) in (2), parameterized with σ^2^ = 0.1 and *D *= 50, yields μ(**B**) = 0.9994 and μ(**B**)_average = 0.9917 (Methods and Supplementary Material 2).

#### Segmentation of the color microscopic image of unstained cryosections of mouse fatty liver

The EKM-NMF_L0 algorithm was tested on the segmentation of images of unstained cryosections of mouse fatty liver (Figures S5a and S6a). Subsequent cryosections were further colored by H&E (Figures S5b and S6b) and by specific Sudan 3 dye (Figures S5c and S6c). Sudan 3 was used to identify the fat storage granules (colored in orange) in the liver cells. Compared to the Sudan 3-stained section (Figure S5c), the EKM-NMF_L0 algorithm successfully discriminated the fatty vacuoles, which are shown in yellow (Figure S5d). Moreover, the EKM-NMF_L0 algorithm was able to discriminate blood vessels (red), sinusoids (sky blue), reticular fibers (magenta) and hepatocytes (green) (Figures S6d and S6e) from the image of the unstained specimen shown in Figure S6a, as confirmed by the visual correspondence with the ground truth images obtained by staining the cryosections with H&E (Figure S6b) and Sudan 3 (Figure S6c).

## Discussion

We derived a blind-source separation method that performs factorization of the multichannel image in RKHS with binary {0, 1} and orthogonality constraints imposed on the sources. When applied to the color microscopic images of unstained specimens, the method decreases the correlation between spectral profiles of tissue components present in the specimen, which is expected to enable separation and then digital staining of the tissue components that otherwise appear colorless and almost texture-less when viewed under a light microscope. The ultimate (long term) aim for the development of the proposed method is the substitution of time consuming staining procedure by digital staining. This goal is motivated by the following reasons. Although the staining of specimens in the slide preparation process has been in place for many years to highlight tissue components,^25^,^26^ it also has limitations and negative effects. Thus, substitution by a method that segments an image of unstained specimens and digitally colors the segmented tissue components would result in the following benefits: (*i*) shortening of the slide preparation process; (*ii*) reduction of the variation in diagnosis between histologists; (*iii*) total elimination of the chemical effects on a specimen; (*iv*) elimination of the morphological changes of a specimen; (*v*) simplification of the histological and intra-surgical tissue analysis; (*vi*) significant cost savings; (*vii*) prevention of harm to the user because chemical stains are not used; (*viii*) discrimination of several tissue components present in the same section of the specimen; and (*ix*) the ability to use the same section of a specimen for more than one analysis.

However, because segmentation of the images of unstained specimens is a very difficult image processing problem, the goal of the present study was to validate, on the proof-of-concept level, the capability of the developed method to separate tissue components present in unstained specimens related to clinically relevant problems. Thus, segmentation has been performed on images of unstained specimens of human hepatocellular carcinoma, human liver with metastasis from colon and gastric cancer, and segmentation of tissue components from the image of unstained specimens of mouse fatty liver. This has been performed approximately correct using an RGB image acquisition system with 8-bits per pixel encoding of each monochromatic image. The Matlab implementation of the proposed method executed segmentation in approximately 250 seconds, whereas implementation on graphical processing units can bring a 10- to 100-fold increase in speed^27^,^28^,^29^, which could make the proposed method suitable for applications such as intraoperative (frozen section) tissue analysis. In that scenario, it is necessary for segmentation and digital staining to be executed quickly. The proposed method could be combined with the images of H&E stained specimens, which have deteriorated quality due to different types of tissue preparation (cryopreservation). Because the proposed method performs segmentation, it can be used for demarcation as well^30^. Arguably, tissue components segmented by the proposed method can be used for texture-based feature extraction^31^ and training of predictive models for computer assisted diagnoses. However, conjectured applications of the proposed method were out of the scope of the current study. It is clear that the performance of the proposed method is limited by the initial contrast (spectral similarity) between the histological structures present in the unstained specimen. This limitation can be improved if, instead of the currently used RGB image acquisition system with 8-bits per pixel encoding of each monochromatic image, a multispectral image acquisition system is used. In this regard, we mention the multispectral imaging systems^32^,^33^ that were used to digitally stain H&E stained images^32^ or unstained images^33^. In particular, a multispectral imaging system with 5-nm spectral resolution comprising 63 monochromatic images encoded with 16-bits per pixel has been used in ref. 32. Such multispectral imaging of unstained specimens will have significantly increased initial contrast (measured by mutual coherence) in comparison with the RGB image of the same specimen. Thus, a combination of the proposed segmentation method and the described multispectral imaging system can substitute for staining in many relevant scenarios in pathology and/or cytology. Furthermore, provided that the SNR is high enough, a decrease of the variance of the Gaussian kernel in EKM can also decrease the correlation between the spectral profiles of low-contrast components (Table 1 and Figures S1 and S2), i.e., denoising of the image of the unstained specimen with an advanced algorithm^34^ could further improve the capability of the proposed method to separate low-contrast histological structures.

## Methods

### Derivations and proofs

We describe the representation of the multichannel RGB color microscopic image by a linear mixture model (1) (Supplementary Materials 1). We discuss how the high correlation between the spectral profiles of histological structures present in the image of the unstained specimen disables the sparseness-constrained factorization algorithms to yield results that are useful for visual interpretation, i.e., to separate healthy and disease tissues (Supplementary Materials 2). We prove that the EKM-based mapping of color images represented by the linear mixture model (1) remains invariant with respect to binary {0, 1} and orthogonal source components (Supplementary Materials 3). We also show that EKM-based mapping of the color images of unstained specimens yields multichannel images with an increased number of non-physical color bands and with significantly less correlated spectral profiles of the same histological structures (Supplementary Materials 3). Furthermore, we describe the sparseness-constrained nonnegative matrix factorization methods used to extract the histological structures from the EKM-mapped color image of the unstained specimen (Supplementary Materials 4). Finally, we perform extensive benchmarks on the synthetic image (Fig. 1 and S1) and the experimental images of unstained specimens (Figs 2 to 4 and S2 to S6).

### Estimation of the correlations between the spectral profiles of tissue components

We write (1) at some arbitrary pixel position:


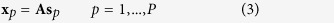


where 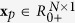 and 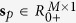. Within this study, it is assumed that sources (tissue components) are orthogonal and binary:


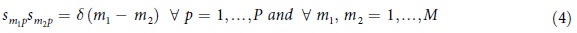


An important implication of (4) is that (3) can be written as:


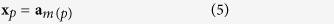


where *m*(*p)*∈{1,_…_, M} denotes the index of the source that is present at pixel *p*. Thus, by virtue of (5), and by knowing the spatial locations of particular histological structures (they can be inferred from the H&E stained image shown in Figures 2e, 3d and/or 4d), spectral profiles 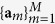 of the tissue components can be estimated from the original image **X**. Afterward, the worst case correlation, i.e., mutual coherence μ(**A**), and the average correlation between 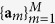, μ_average(**A**), are calculated as:


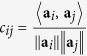



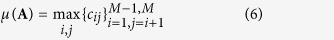



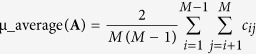


where 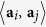 stands for the inner product between **a**_*i*_and **a**_*j*,_ and 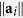 stands for the 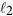-norm of **a**_*i*_.Values of *μ*(**A**) and/or *μ_*average(**A**) “very close” to 1 drastically reduce the possibility of separating corresponding tissue components.

### Immunohistochemistry of human and animal specimens

Paraffin-embedded samples of human liver tissue were sectioned (at 5 μm) using a microtome (Leica RM2125RTS). After deparaffinization and rehydration (by xylene and a series of ethyl alcohol concentrations), antigen retrieval was performed by citrate buffer pH 6.0 at sub-boiling temperature. The section was then photographed with a microscope camera followed by staining with H&E. Subsequent sections of the same tissue sample were further stained using immunohistochemistry (IHC). IHC is a method for localizing specific antigens in tissues or cells based on antigen-antibody recognition. It seeks to exploit the specificity provided at the light microscopic level. An antibody is a molecule that has the property of combining specifically with a second molecule, termed the antigen. Antigen-antibody recognition is based on the three-dimensional structure of the protein or antigen, which is a critical issue in this type of staining. In this study, we used several specific primary antibodies (Hepatocyte clone OCH1E5, CDX2 clone DAK-CDX2, LCA clone 2B11 + PD7/26, and CK20 clone Ks20.8, all from Dako, Denmark)^35^ and stained using the Dako Envision System (Denmark). CDX-2 is a homebox gene that encodes the CDX-2 protein, which is a transcription factor expressed in the nuclei of intestinal epithelial cells. CDX2 is also used in diagnostic surgical pathology as a marker for gastrointestinal differentiation, especially colorectal differentiation. Hepatocyte Paraffin 1 (Hep Par 1) recognizes the mitochondrial antigen of hepatocytes and determines the hepatocellular origin of cells. It is also positive in hepatocellular carcinoma and can differentiate HCC from cholangiocarcinoma or metastases to the liver. Keratin 20, often abbreviated CK20, is a protein that is encoded by the *KRT20* gene in humans. It is a major cellular protein of mature enterocytes and goblet cells and is specifically found in the gastric and intestinal mucosa. In IHC, antibodies to CK20 can be used to identify a range of adenocarcinomata arising from epithelia that normally contain the CK20 protein. For example, the protein is commonly found in colorectal cancer. LCA (CD45) is a family of single chain transmembrane glycoproteins. CD45 is exclusively expressed in haematolymphoid cells: precursor cells and mature B- and T-lymphocytes, granulocytes, monocytes/histiocytes and interdigitating reticulum cells and follicular dendritic cells.

CBA mice of both sexes (3–4 weeks of age) were obtained from the Animal Facility of Rudjer Boskovic Institute. The mice were fed a high-fat diet containing 58% energy from fats, 24% from carbohydrates and 18% from proteins (Mucedola, Italy) during a 15-week period. Food and water was provided *ad libitum*. The animals were housed in standard conditions: 3 mice per cage, at 22 °C and 50–70% humidity, with a 12 h light and 12 h darkness cycle. Body weight and food intake were monitored once a week. At the end of the study period, the mice were killed by CO_2_ gas. The livers were fixed in 4% p-formaldehyde (Fluka) in PBS containing 0.1% picric acid for at least 4 h, washed in PBS and preserved by immersion in 20% sucrose in PBS overnight. The liver tissue was then frozen in OCT compound (Sakura Finetek, USA) and cryosectioned at 7 μm. One liver section was stained by H&E (after acquisition of an image under a light microscope), and a subsequent section was stained by Sudan 3 (1% Sudan 3 solution in 70% ethyl alcohol, Kemika, Croatia).

### Implementation details

Studies on the numerical and experimental data reported above were executed on a personal computer running the Windows 64-bit operating system with 64 GB of RAM using an Intel Core i7-3930 K processor and operating with a clock speed of 3.2 GHz. The Matlab (Matlab 2012b, MathWorks) environment was used for programming. Implementation of the NMF_L0 algorithm in the MATLAB^®^ script language (The MathWorks, Inc., Natick, MA) is freely available at “Peharz, R., NMF with l0-sparseness constraints: https://www.spsc.tugraz.at/tools/nmf-l0-sparseness-constraints (Date of access: 5/3/2015)”. Implementation of the NMU algorithm in the MATLAB^®^ script language is freely available at "Gillis, N., Nonnegative matrix underapproximation (NMU): https://sites.google.com/site/nicolasgillis/code - Global NMU (Date of access: 5/3/2015). Images were acquired under an Olympus BX51 fluorescence light microscope with a DP50 camera, UPPLANFL objective with 40 × magnification and numerical aperture 0.75, an objective lens with magnification of 10 × (that yields an overall magnification of 400×), 0.45 μm spatial resolution, and illumination between 480 nm and 620 nm. The size of the sample was 459.26 μm × 344.2 μm. Viewfinder Lite 1.0 image acquisition software (analySIS^®^) with 8-bit resolution per monochromatic (spectral) image was used to capture the RGB image with a size of 2074 × 2776 pixels. Before segmentation, the acquired images were downsampled to the size of 1037 × 1388 pixels. Thus, the area covered by one pixel amounts to 0.1098 μm^2^. Because the spatial resolution of the microscope is 0.45 μm, the assumption that each pixel is occupied by only one tissue component is justified. The segmentation of each image by the EKM-NMF_L0 algorithm took approximately 250 seconds.

### Studies involving human subjects and animals

The methods in the study were carried out in accordance with approved guidelines by Clinical Hospital Dubrava Ethics Committee (October 10, 2013) and the Bioethics Committee of the Ruđer Bošković Institute (BP-2290/2-2012). Because of its retrospective nature, informed consent was not required from human subjects.

## Additional Information

**How to cite this article**: Kopriva, I. *et al* Unsupervised segmentation of low-contrast multichannel images: discrimination of tissue components in microscopic images of unstained specimens. *Sci. Rep* **5**, 11576; doi: 10.1038/srep11576 (2015).

## Supplementary Material

Supplementary Information

## Figures and Tables

**Table 1:** Variance *σ*^2^ of the Gaussian kernel based EKM as a function of the per-spectral-channel SNR.

SNR [dB]	SNR ≥ 29	18 ≤ SNR ≤ 28	17 ≤ SNR ≤ 14
*σ*^2^	0.001	0.01	0.1

**Table 2:** The mixing matrix A in (1).

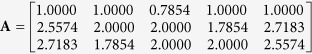

## References

[b1] Gonzalez R. C. & Woods R. E. in Digital Image Processing (Prentice Hall, 2007).

[b2] Jain V., Seung S. H. & Turaga S. C. Machines that learn to segment images: a crucial technology for connectomics. Curr. Opin. Neurobiol. 20, 653–666 (2010). 20801638 10.1016/j.conb.2010.07.004 PMC2975605

[b3] Marr D. & Hildredth E. Theory of edge detecion. Proc. Royal Soc. London Series B Biol. Sci. 207, 187–217 (1980). 10.1098/rspb.1980.0020 6102765

[b4] Geman S. & Geman D. Stochastic relaxation, Gibbs distributions, and the Bayesian restoration of images. IEEE Trans. Pattern Anal. Mach. Intell. 6, 721–741 (1984). 22499653 10.1109/tpami.1984.4767596

[b5] Boykov Y., Veksler O. & Zabih R. Fast Approximate Energy Minimization via Graph Cuts. IEEE Trans. Pattern Anal. Mach. Intell. 23, 1222–1239 (2001).

[b6] Kass M., Witkin A. & Terzopoulos D. Snakes: Active contour models. Int. J. Comput. Vis. 1, 321–331 (1988).

[b7] Osher S. & Fedkiw R. P. Level Set Methods: An Overview and Some Recent Results. J. Comput. Phys. 169, 463–502 (2001).

[b8] Chitade A. Z. & Katiyar S. K. Colour Based Image Segmentation Using K-Means Clustering. Int. J. Eng. Sci. Tech. 2, 5319–5325 (2010).

[b9] Comon P. Independent Component Analysis: a new concept? Sig. Proc. 36, 287–314 (1994).

[b10] Lee. D.D. & Seung S.H. Learning the parts of objects by nonnegative matrix factorization. Nature 401, 788–791 (1999). 10548103 10.1038/44565

[b11] Bofill P. & Zibulevsky M. Underdetermined blind source separation using sparse representations. Sig. Proc. 81, 2353–2362 (2001).

[b12] Handbook of Blind Source Separation (eds Comon P. .) (Academic Press, 2010).

[b13] Li Y., Cichocki A. & Zhang L. Blind Separation and Extraction of Binary Sources. IEICE Trans. Fund. E86, 580–589 (2003).

[b14] Diamantaras K. I. A clustering approach for the blind separation of multiple finite alphabet sequences from a single linear mixture. Sig. Proc. 86, 877–891 (2006).

[b15] Diamantaras K.I, Papadimtriou T. & Vranou G. Blind separation of multiple binary sources from one nonlinear mixture. Paper presented at *IEEE International Conference Acoustics, Speech and Signal Processing (ICASSP)*, Prague, IEEE (10.1109/ICASSP.2011.5946742) (2011, May, 22-27).

[b16] Castella M. Inversion of Polynomial Systems and Separation of Nonlinear Mixtures of Finite-Alphabet Sources. IEEE Trans. Sig. Proc. 56, 3905–3917 (2008).

[b17] Schölkopf B. & Smola A. in Learning with kernels (MIT Press, 2002).

[b18] Kopriva I., Jerić I., Filipović M. & Brkljačić L. Empirical Kernel Map Approach to Nonlinear Underdetermined Blind Separation of Sparse Nonnegative Dependent Sources: Pure Components Extraction from Nonlinear Mixtures Mass Spectra. J. Chemometrics 28, 704–715 (2014).

[b19] Peharz R. & Pernkopf F. Sparse nonnegative matrix factorization with *ℓ*^0^-constraints. Neurocomputing 80, 38–46 (2012). 22505792 10.1016/j.neucom.2011.09.024 PMC3312776

[b20] Gillis N. & Glineur F. Using underapproximations for sparse nonnegative matrix factorization. Pattern Rec. 43, 1676–1687 (2010).

[b21] Fukunaga K. & Olsen D. R. An Algorithm for Finding Intrinsic Dimensionality of Data IEEE Trans. on Computers C-20, 176–183 (1971).

[b22] Stoica P. & Selen Y. Model-Order Selection. IEEE Sig. Proc. Mag. 21, 36–47 (2004).

[b23] Malinowski E. R. in Factor Analysis in Chemistry (John Wiley & Sons, 2002).

[b24] McCann T. M. Images as occlusions of random textures: A framework for segmentation. IEEE Trans. Image Proc. 23, 2033–2046 (2014). 10.1109/TIP.2014.2307475 24710403

[b25] Crawford J. M. & Burt A. D. in Pathology of the liver (eds Burt A. D. .) Ch. 1, 1–77 (Elsevier, 2011).

[b26] Sertel O. Histopathological image analysis using model-based intermediate representations and color texture: Follicular lymphoma grading. J. Sig. Proc. Syst. 55, 169–183 (2009).

[b27] Preibisch S. Efficient Bayesian-based multiview deconvolution. Nat. Methods 11, 645–648 (2014). 24747812 10.1038/nmeth.2929 PMC4153441

[b28] Ikeda K., Ino F., & Hagihara K. Efficient Acceleration of Mutual Information Computation for Nonrigid Registration Using CUDA. IEEE J. Biomed. Health. Inf. 18, 956–968 (2014). 10.1109/JBHI.2014.2310745 24808228

[b29] Eklund A., Dufort P., Forsberg D., & LaConte S. M. Medical image processing on the gpu – past, present and future. Med. Image Anal. 17, 1073–1094 (2013). 23906631 10.1016/j.media.2013.05.008

[b30] Kopriva I., & Peršin A. Unsupervised decomposition of low-intensity low-dimensional multi-spectral fluorescent images for tumour demarcation. Med. Image Anal. 13, 507–518 (2009). 19282233 10.1016/j.media.2009.02.002

[b31] Liu L., & Fieguth P. W. Texture classification from random features. IEEE Trans. Pattern Anal. Mach. Intell. 34, 574–586 (2012). 21768653 10.1109/TPAMI.2011.145

[b32] Bautista P. A., & Yagi Y. Digital simulation of staining in histopathology multispectral images: enhancement and linear transformation of spectral transmittance. J. Biomed. Opt. 17(5), 056013 (2012). 22612136 10.1117/1.JBO.17.5.056013

[b33] Bautista P. A., Abe T., Yamaguchi M., Yagi Y., & Ohyama N. Digital staining of unstained pathological tissue samples through spectral transmittance classification. Opt. Rev. 12, 7–14 (2005). 10.1016/j.compmedimag.2005.09.003 16269238

[b34] Mairal J., Elad M., & Sapiro G. Sparse Representation for Color Image Restoration. IEEE Trans. Image Process. 17, 53–69 (2008). 18229804 10.1109/tip.2007.911828

[b35] Dabbs D. J. in Diagnostic immunohistochemistry (Saunders, 2013).

