# Supplementary material for: Unsupervised segmentation of low-contrast multichannel images: discrimination of tissue components in microscopic images of unstained specimens
Source: Sci Rep. 2015 Jun 23;5:11576. doi: 10.1038/srep11576 (PMC4477329; doi:10.1038/srep11576)
Supplement: Supplementary Information [file srep11576-s1.pdf]

Unsupervised segmentation of low-contrast multichannel images: discrimination of tissue components in microscopic images of unstained specimens

Ivica Kopriva, Marijana Popović Hadžija, Mirko Hadžija, Gorana Aralica

Supplementary File	Title
**Figure S1**	Segmentation results of synthetic color image by means of EKM-NMU method for various combinations of EKM parameters: D and σ^2, and SNR values
**Figure S2**	Segmentation results of the images of sections of a specimen of human hepatocellular carcinoma
**Figure S3**	Segmentation results of the images of sections of a specimen of human liver with metastasis from colon cancer
**Figure S4**	Segmentation results of the images of sections of a specimen of human liver with metastasis from gastric cancer
**Figure S5**	Segmentation results of the images of sections of a specimen of mouse fatty liver
**Figure S6**	Segmentation results of the images of sections of a specimen of mouse fatty liver composed of hepatocytes, blood vessel, reticular fibers and sinusoids
**Figure S7**	Amount of sparseness as a function of mutual coherence imposed by uniqueness condition without noise (5), stability condition (8) and uniqueness condition (9) for basis pursuit denoising algorithm
**Supplementary Material 1**	Linear mixture model-based representation of multichannel image
**Supplementary Material 2**	Mutual coherence of basis (mixing) matrix, spectral diversity and uniqueness of multichannel image decomposition
**Supplementary Material 3**	Empirical kernel map-based nonlinear mapping of multichannel image
**Supplementary Material 4**	Sparseness and non-negativity constrained factorization

SUPPLEMENTARY FIGURES

FIGURE S1. Segmentation results of synthetic color image by means of EKM-NMU method for various combinations of EKM parameters: *D* and σ^2, and SNR values.

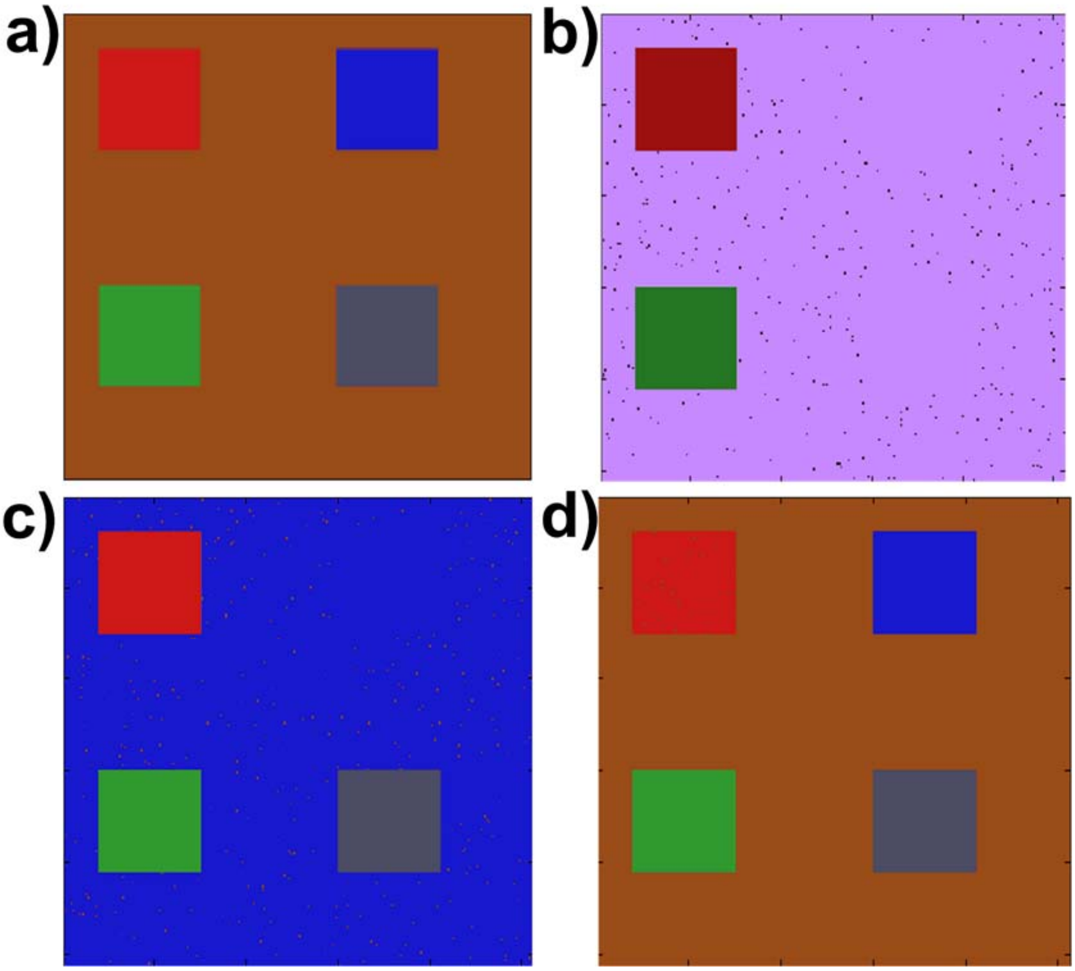

Figure S1. *Color coded results of segmentation of synthetic image shown in Figure 1a*. First, encoding coefficients $\mathbf{S} \in \mathbb{R}_{0+}^{M \times P}$ were estimated by means of EKM-NMU algorithm with D=20. Afterwards, encoding coefficients are mapped column- (pixel) wise: $\left\{\mathbf{s}_p \mapsto \bar{\mathbf{s}}_p\right\}_{p=1}^{P}$, such that

$$\bar{s}_{mp} = \begin{cases} 1 & for \;\; m = \arg\max_i \left\{s_{ip}\right\}_{i=1}^{M} \\ 0 & otherwise. \end{cases}$$

. Colors are assigned to folded (2D) equivalents of $\left\{\bar{\mathbf{s}}_m\right\}_{m=1}^{M}$ according to color coded ground truth image shown in (a). Results are shown for combination of variance of Gaussian kernel σ^2 and highest SNR value in dB for which obtained result differs from color coded ground truth. (b) $\sigma^2=10^{-3}$, SNR=28 dB. (c) $\sigma^2=10^{-2}$, SNR=17 dB. (d) $\sigma^2=10^{-1}$, SNR=13 dB. Thus, lowest SNR values for which correct segmentation results are still obtained are given respectively as 29 dB, 18 dB and 14 dB. Hence, by increasing variance of Gaussian kernel in EKM-based mapping noise robustness can be increased significantly.

FIGURE S2. Segmentation results of the images of sections of a specimen of human hepatocellular carcinoma

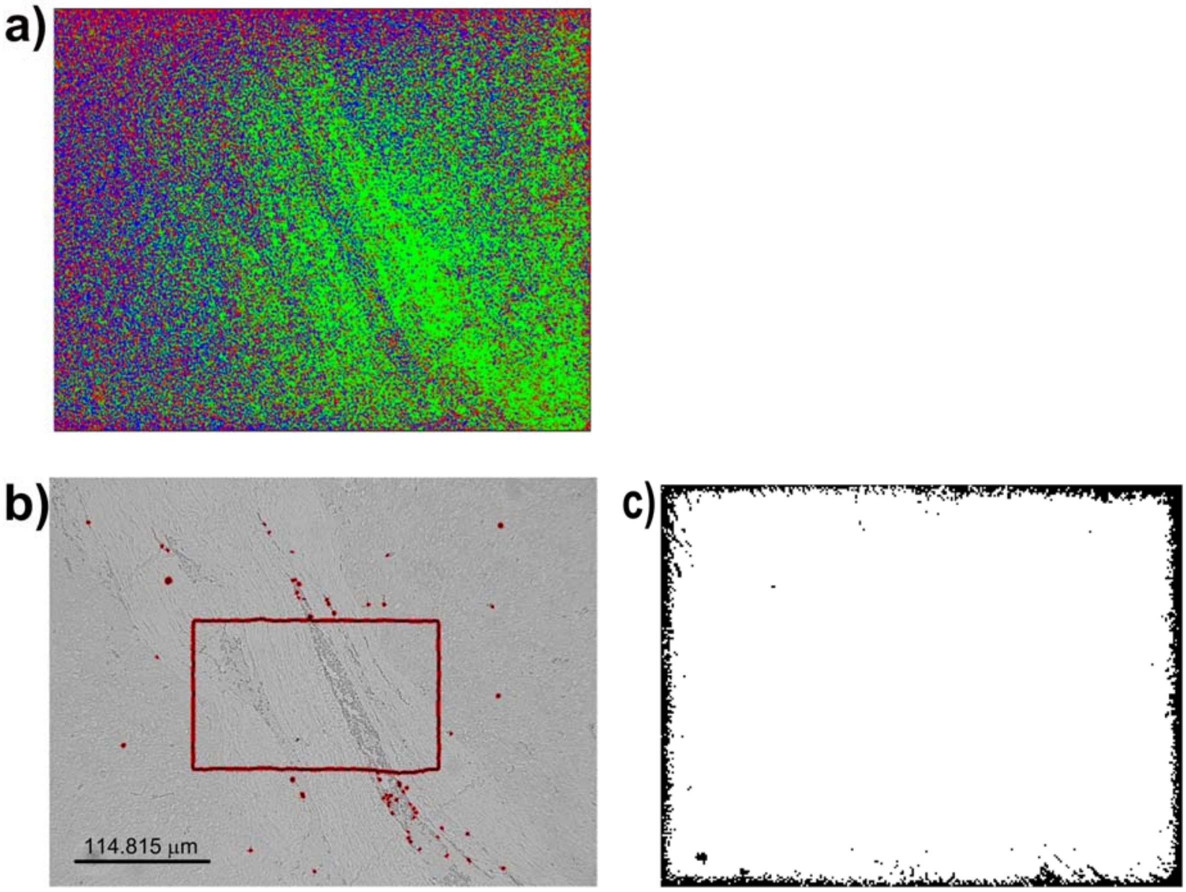

d)

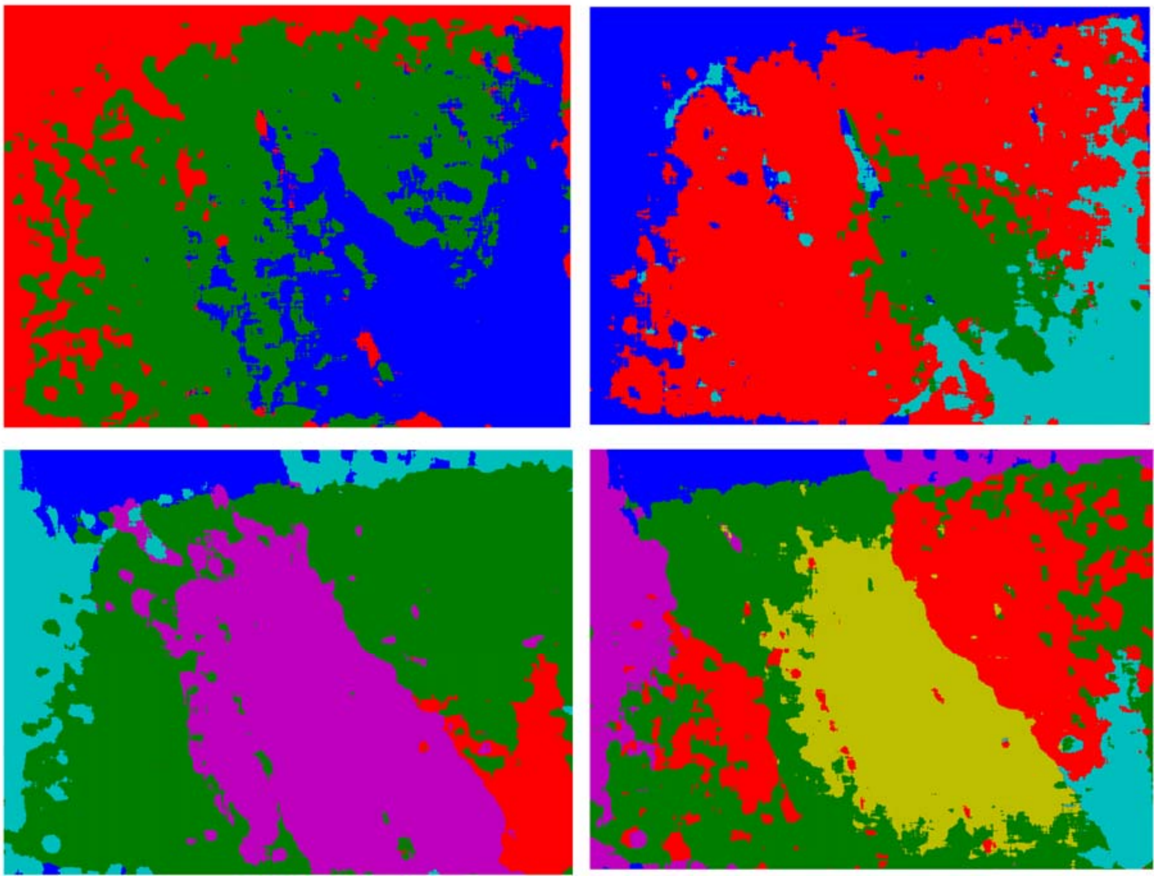

e)

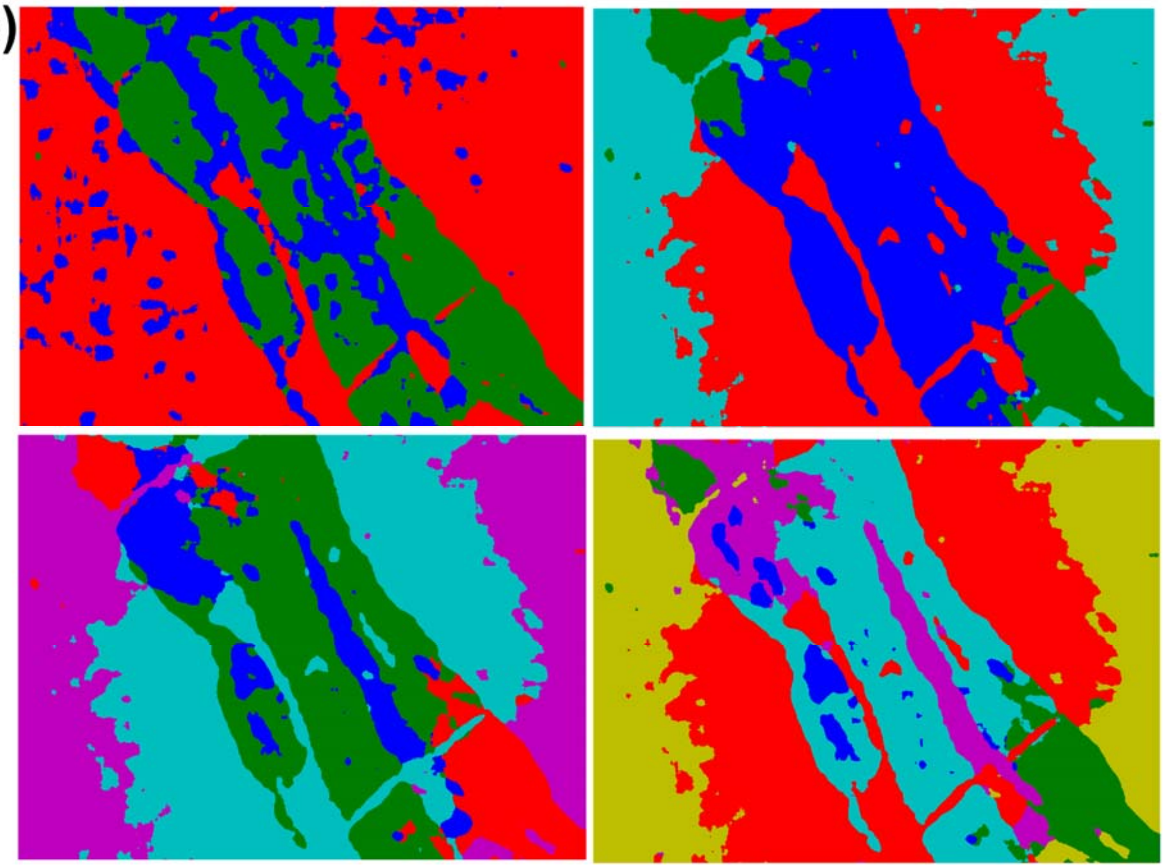

f)

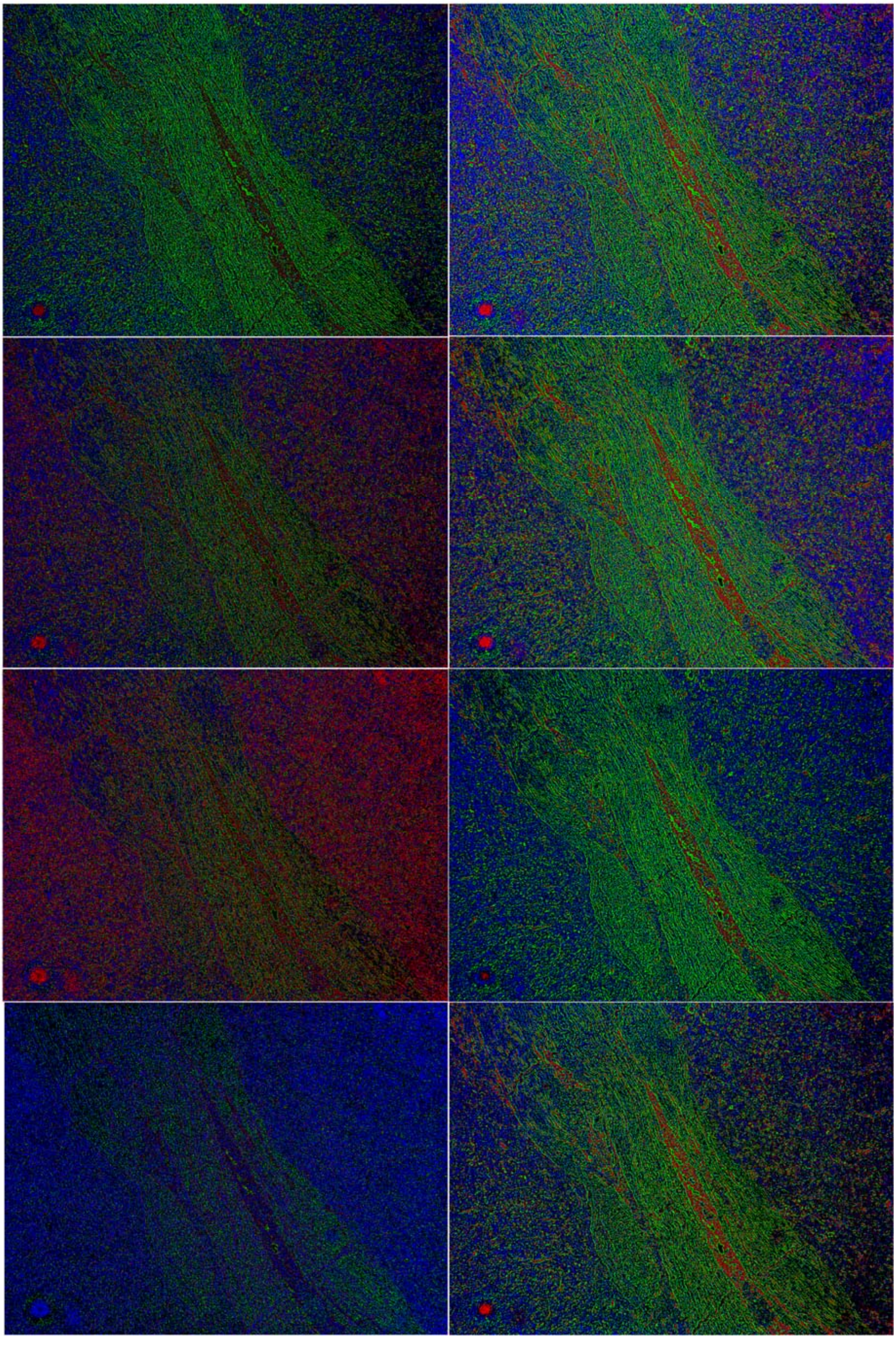

Figure S2. *Some segmentation results of images of sections of a specimen human hepatocellular carcinoma.* (a) segmentation result of the image of unstained section, shown in figure 2a, obtained by *k-means* clustering in the CIE $L^*a^*b^*$ color space[1]. (b) segmentation result on grayscale version of figure 2a by means of the geometric active contour (GAC)[2] method with new distribution metric[3,4] after 6000 iterations. (c) segmentation result on figure 2a by means of active contour method for vector images[5,6]. (d) segmentation result of image of unstained section, shown in figure 2a, obtained by ORTSEG algorithm[7]. The number of textures (histological structures) assumed to be present in figure 2a is from left to right 3 and 4 (top row), and 5 and 6 (bottom row). (e) segmentation results of image of section stained by H&E, shown in figure 2e, obtained by ORTSEG algorithm. The number of textures (histological structures) assumed to be present in figure 2e is from left to right 3 and 4 (top row), and 5 and 6 (bottom row). (f) color coded (digitally stained) image of segmentation results of the image of unstained section, shown in figure 2a, obtained by EKM-NMF_L0 algorithm: blue: region of hepatocellular carcinoma, red: blood vessel, green: tumor fibrotic capsule. Variance σ^2 of Gaussian kernel equals 0.01 in left column and 0.1 in right column. Dimensionality D of EKM-induced vector space equals respectively from top to bottom: 150, 100, 50 and 25.

FIGURE S3. Segmentation results of the images of sections of a specimen of human liver with metastasis from colon cancer

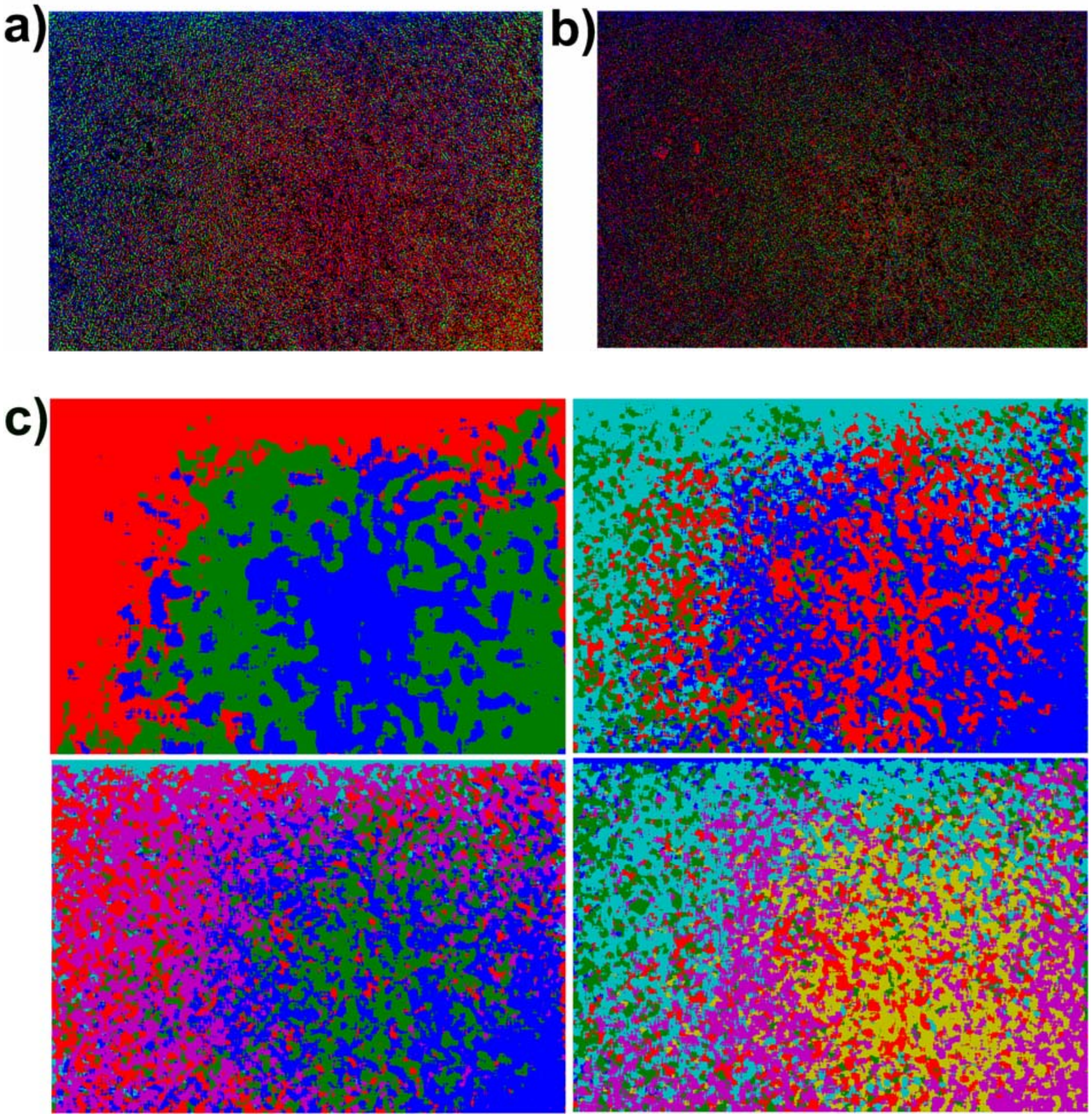

d)

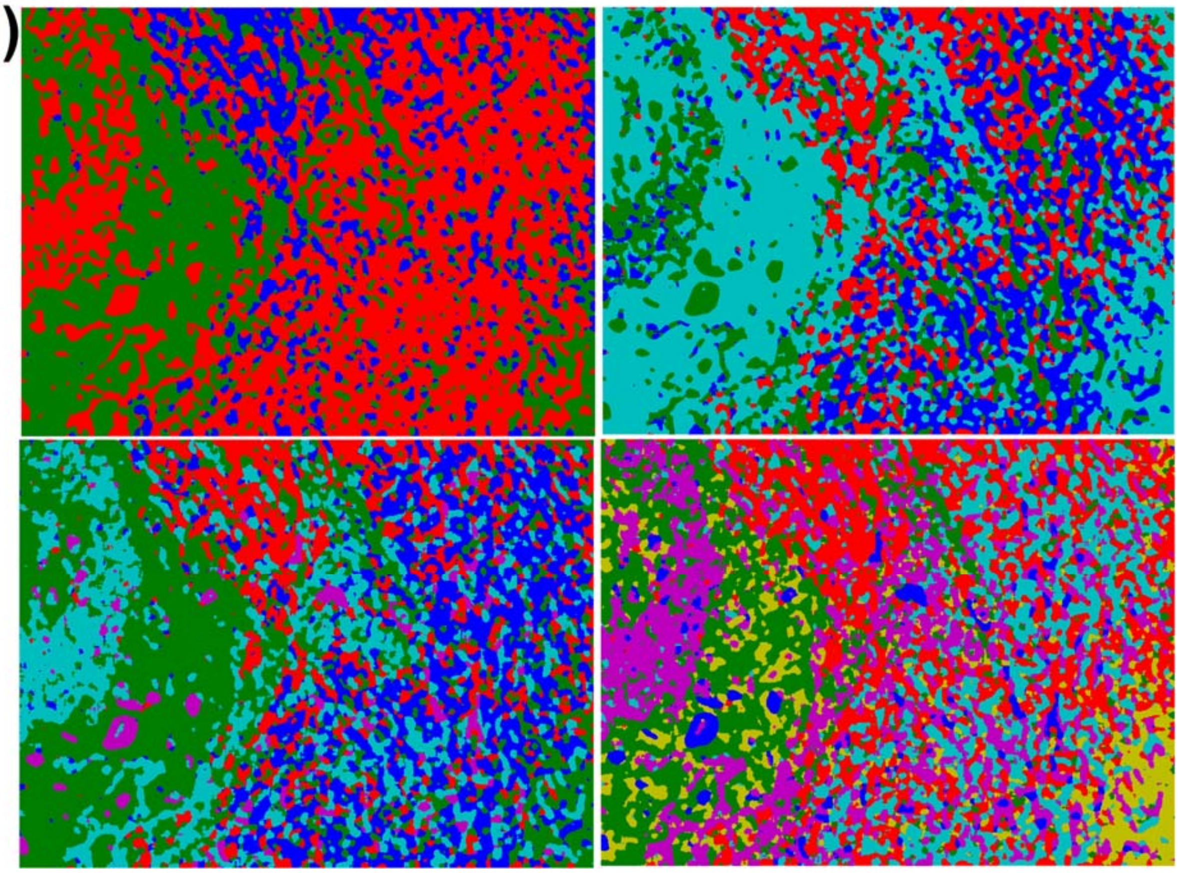

Figure S3. *Some segmentation results of images of sections of a specimen of human liver with metastasis from colon cancer.* (a) segmentation result of the image of unstained section, shown in figure 3a, obtained by *k-means* clustering in the CIE $L^*a^*b^*$ color space. (b) segmentation result of the image of unstained section, shown in figure 3a, obtained by NMF_L0 algorithm[8]: blue: metastatic cells from colon cancer, red: border area between tumor and liver tissue, green: hepatocytes. (c) segmentation result of image of unstained section, shown in figure 3a, obtained by ORTSEG algorithm. The number of textures (histological structures) assumed to be present in figure 3a is from left to right 3 and 4 (top row), and 5 and 6 (bottom row). (d) segmentation results of image of section stained by H&E, shown in figure 3d, obtained by ORTSEG algorithm. The number of textures (histological structures) assumed to be present in figure 3d is from left to right 3 and 4 (top row), and 5 and 6 (bottom row).

FIGURE S4. Segmentation results of the images of sections of a specimen of human liver with metastasis from gastric cancer

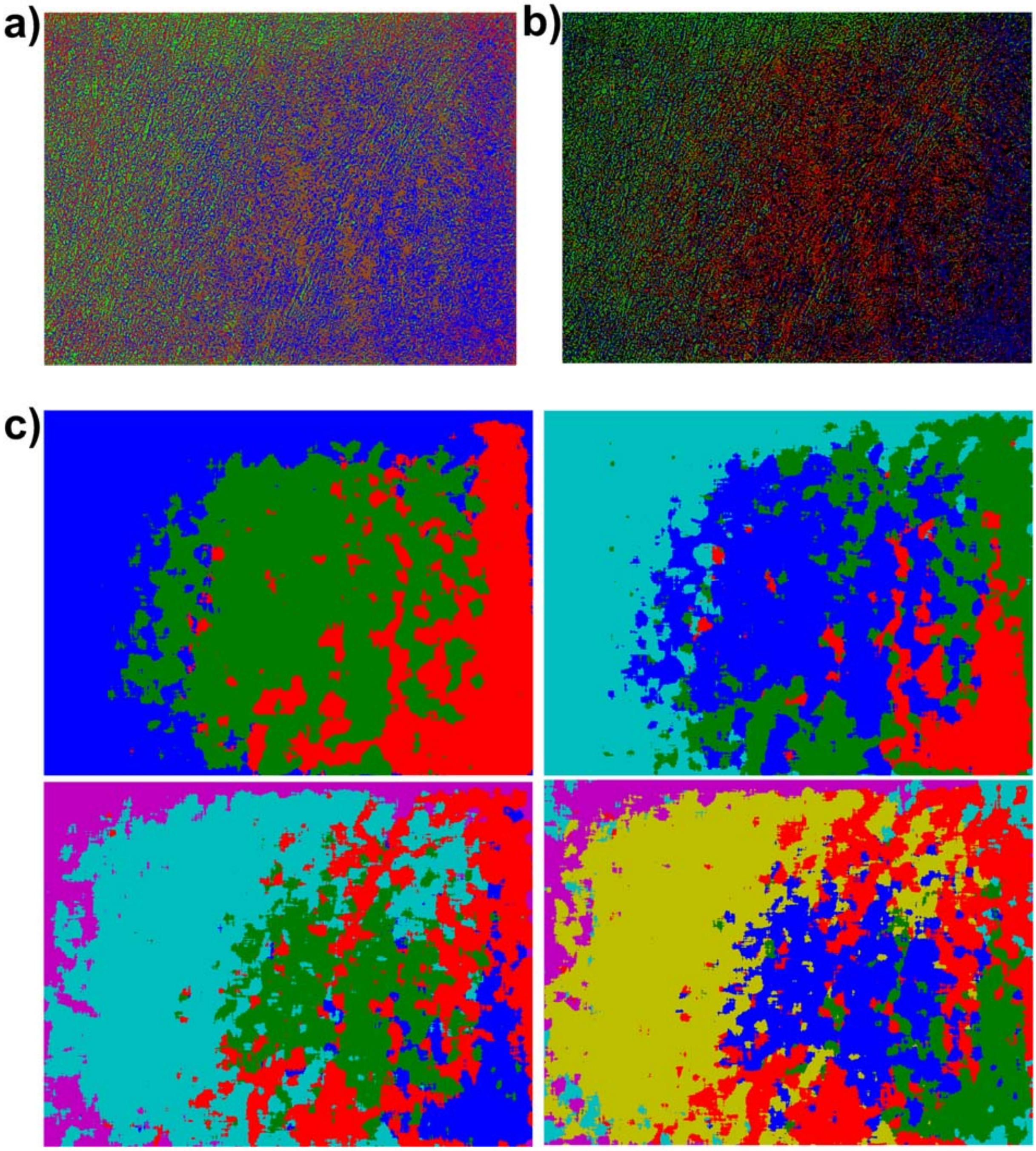

d)

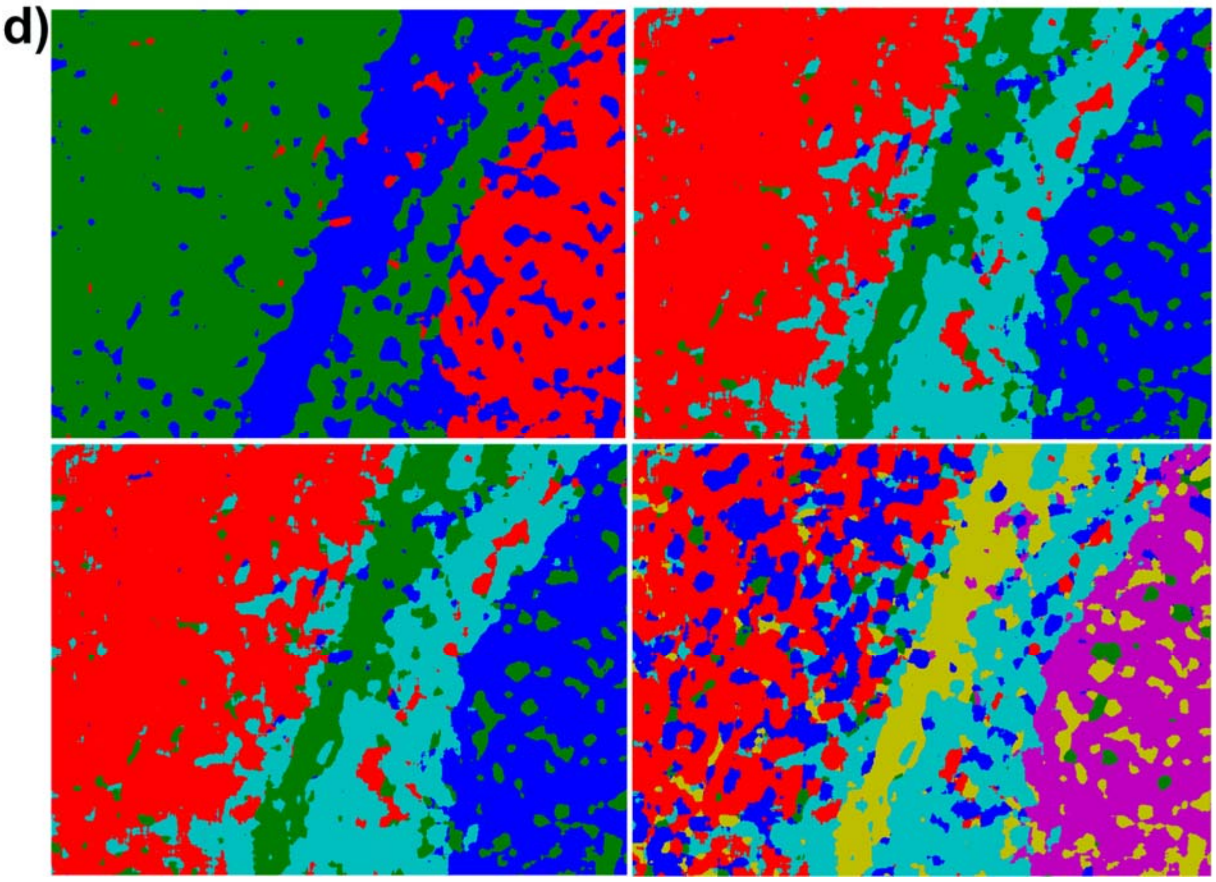

Figure S4. *Some segmentation results of images of sections of a specimen of human with metastasis from gastric cancer.* (a) segmentation result of the image of unstained section, shown in figure 4a, obtained by *k-means* clustering in the CIE $L^*a^*b^*$ color space. (b) segmentation result of the image of unstained section, shown in figure 4a, obtained by NMF_L0 algorithm. Blue: metastatic cells of gastric cancer, red: border area of inflammation, green: hepatocytes. (c) segmentation result of image of unstained section, shown in figure 4a, obtained by ORTSEG algorithm. The number of textures (histological structures) assumed to be present in figure 4a is from left to right 3 and 4 (top row), and 5 and 6 (bottom row). (d) segmentation results of image of section stained by H&E, shown in figure 4d, obtained by ORTSEG algorithm. The number of textures (histological structures) assumed to be present in figure 4d is from left to right 3 and 4 (top row), and 5 and 6 (bottom row).

FIGURE S5. Segmentation result of the images of sections of a specimen of mouse fatty liver

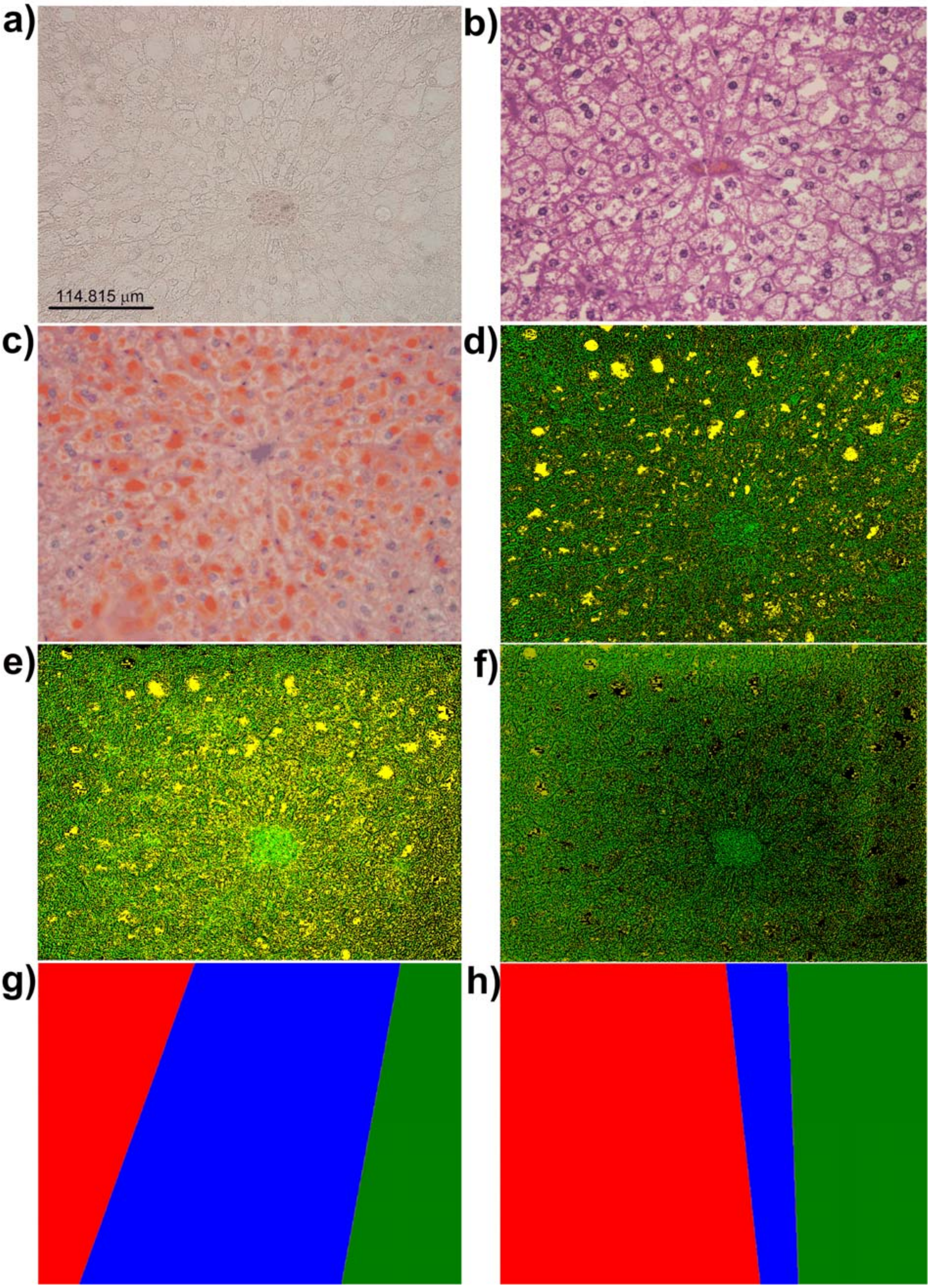

Figure S5. *Segmentation result of images of a specimen of mouse fatty liver.* (a) RGB color microscopic image of unstained section. (b) "ground truth - different section" RGB color microscopic image of the section stained by H&E. (c) "ground truth - different section" RGB color microscopic image of the section stained by SUDAN 3. (d) color coded (digitally stained) image of the segmentation result obtained by EKM-NMF_L0 algorithm (Gaussian kernel variance=0.1 and D=50): yellow: vacuoles, green: liver parenchyma. (e) color coded (digitally stained) image of the segmentation result obtained by *k-means* clustering in the CIE $L^*a^*b^*$ color space: yellow: vacuoles, green: liver parenchyma. (f) color coded (digitally stained) image of the segmentation result obtained by NMF_L0 algorithm: yellow: vacuoles, green: liver parenchyma. (g) color coded (digitally stained) image of the segmentation result of the image of unstained section (a) and obtained by ORTSEG algorithm. The number of textures (histological structures) assumed to be present in (a) is 3. (h) color coded (digitally stained) image of the segmentation result of the image of section stained by H&E (b) and obtained by ORTSEG algorithm. The number of textures (histological structures) assumed to be present in (b) is 3.

FIGURE S6. Segmentation results of the images of sections of a specimen of mouse fatty liver composed of hepatocytes, blood vessel, reticular fibers and sinusoids

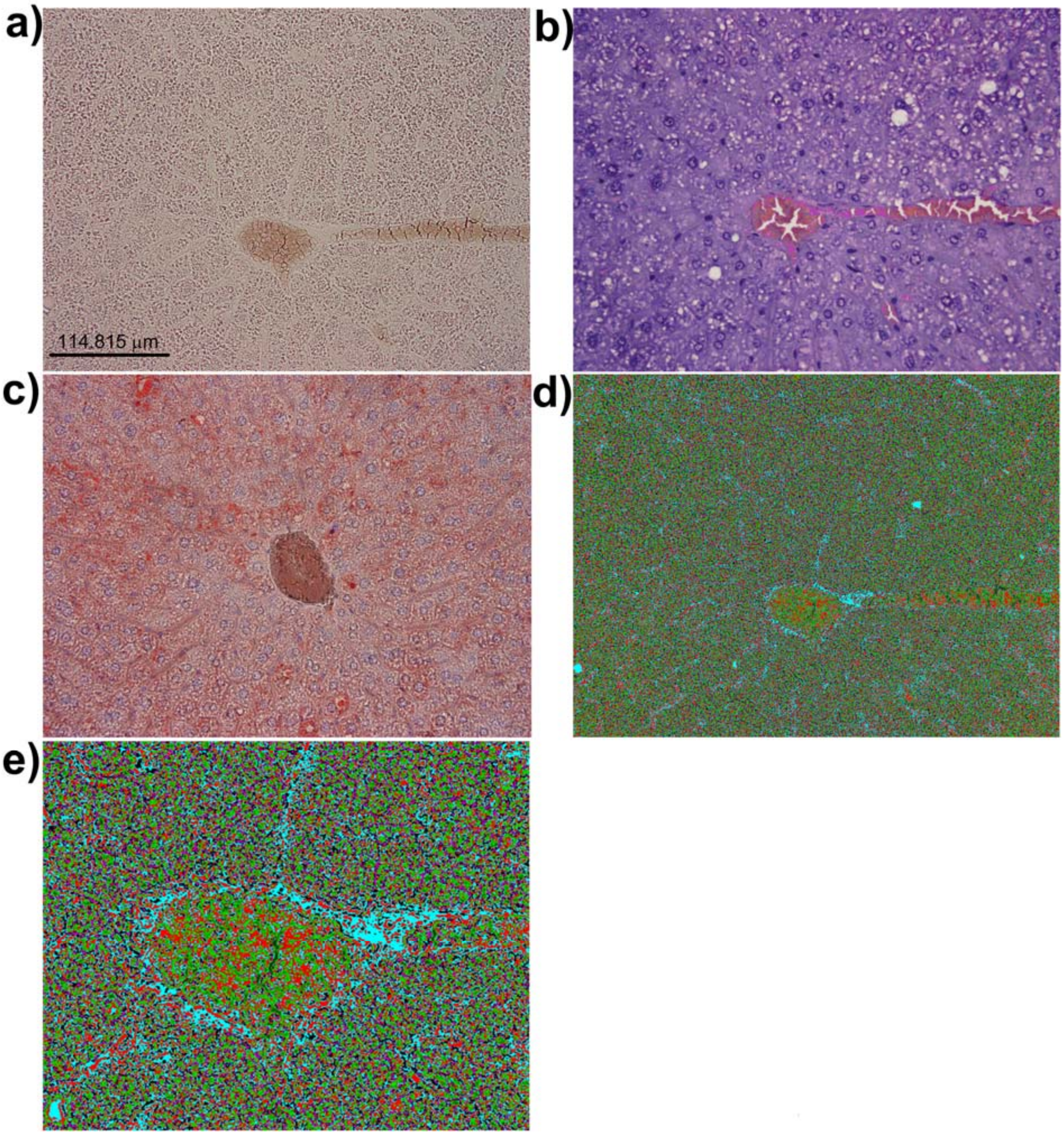

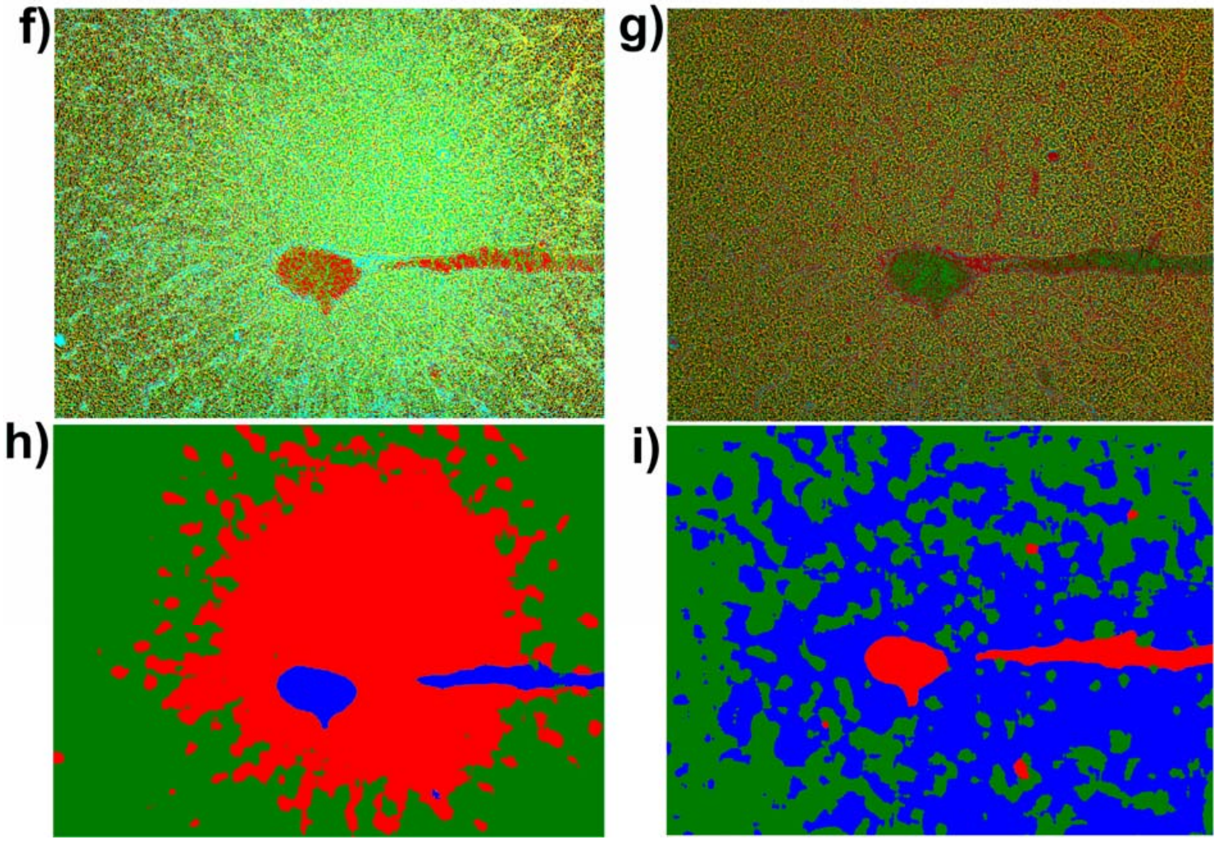

Figure S6. *Some segmentation results of images of sections of a specimen of mouse fatty liver.* (a) RGB color microscopic image of unstained section. (b) "ground truth – different section" RGB color microscopic image of the section stained by H&E. (c) "ground truth - different section" RGB color microscopic image of the section stained by SUDAN 3. (d) color coded (digitally stained) image of the segmentation result obtained by EKM-NMF_L0 algorithm (Gaussian kernel variance=0.1 and D=50): red: blood vessel, sky blue: sinusoids, green: hepatocytes, magenta: reticular fiber. (e) zoomed version of (d). (f) color coded (digitally stained) image of the segmentation result of image of unstained section (a) obtained by *k-means* clustering in the CIE $L^*a^*b^*$ color space: red: blood vessel, sky blue: sinusoids, green: hepatocytes, magenta: reticular fiber. (g) color coded (digitally stained) image of the segmentation result of image of unstained section (a) obtained by NMF_L0 algorithm: red: blood vessel, sky blue: sinusoids, green: hepatocytes, magenta: reticular fiber. (h) color coded (digitally stained) image of the segmentation result of the image of unstained section (a) and obtained by ORTSEG algorithm. Convincing interpretation of results is difficult. The number of textures (histological structures) assumed to be present in (a) is 3. (i) color coded (digitally stained) image of the segmentation result of the image of section stained by H&E (b) and obtained by ORTSEG algorithm. Convincing interpretation of results is difficult. The number of textures (histological structures) assumed to be present in (b) is 3.

FIGURE S7. Amount of sparseness as a function of mutual coherence imposed by uniqueness condition without noise (5), stability condition (8) and uniqueness condition (9) for basis pursuit denoising algorithm.

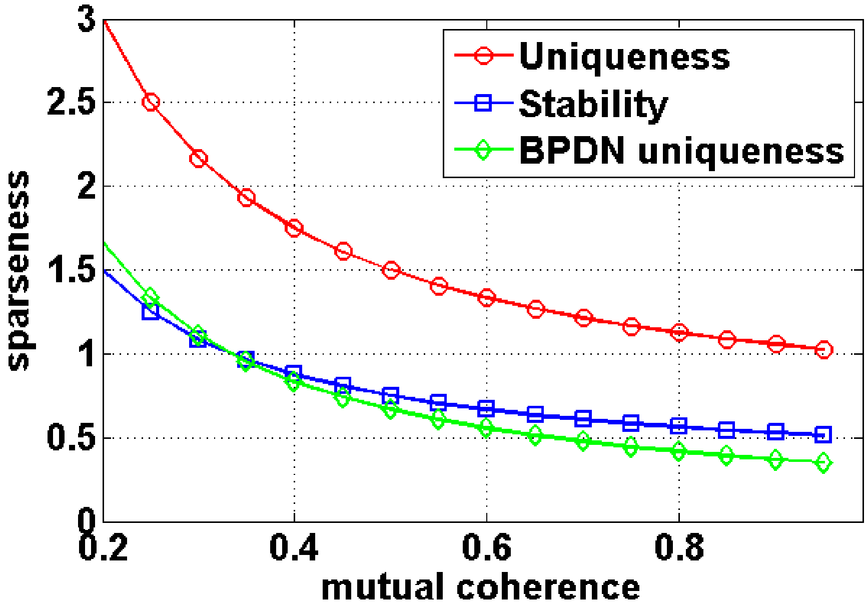

Figure S7. *Amount of sparseness as a function of mutual coherence imposed by various uniqueness/stability conditions*. **Circles**: uniqueness condition without noise (5). **Squares**: stability condition (8). **Diamonds**: uniqueness condition for basis pursuit denoising algorithm (9). For non-overlapping (orthogonal) histological structures sparseness equals $\left\|\mathbf{s}_p\right\|_0 = 1$. Thus, while condition (5) is satisfied even when $\mu(\mathbf{A})\approx 1$ it is seen that approximately $\mu(\mathbf{A})<0.33$ is required to satisfy (8) and (9).

SUPPLEMENTARY MATERIAL 1: LINEAR MIXTURE MODEL-BASED REPRESENTATION OF MULTICHANNEL IMAGE

In multi-channel imaging, such as multispectral/hyperspectral imaging[9,10,11], magnetic resonance imaging[12] and/or multi-phase computed tomography imaging[13], it is customary to represent an image as a weighted linear combination of intensity distributions of objects present in the image:

$$\mathbf{X}=\mathbf{AS} \tag{1}$$

where $\mathbf{X}\in\mathbb{R}_{0^+}^{N\times P} =: \left\{\mathbf{x}_n \in \mathbb{R}_{0+}^{1\times P}\right\}_{n=1}^{N}$ represents multi-channel image comprised of P pixels and symbol =: means *by definition*. Thereby, each row of **X** is a 1D image representation obtained from corresponding channel image by 2D→1D mapping called vectorization. When **X** represents red-green-blue (RGB) microscopic color image number of channels equals N=3 and they correspond with images acquired at wavelengths corresponding with red, green and blue colors. $\mathbf{A}\in\mathbb{R}_{0^+}^{N\times M} =: \left\{\mathbf{a}_m \in \mathbb{R}_{0+}^{N\times 1}\right\}_{m=1}^{M}$ represents basis or mixing matrix comprised column-wise of basis or mixing vectors. In case of multispectral/hyperspectral image mixing vectors represent spectral profiles of the objects present in the image. $\mathbf{S}\in\mathbb{R}_{0^+}^{M\times P} =: \left\{s_{mp} \in \mathbb{R}_{0+}\right\}_{m,p=1}^{M,P}$ represents matrix with the coefficients s_{mp} that encode amount of presence of a component m at a pixel p. Thus, rows of **S**, $\left\{\mathbf{s}_m \in \mathbb{R}_{0+}^{1\times P}\right\}_{m}^{M}$, stand for vectorized partitions that correspond with distinct objects present in the image **X**. In the BSS vocabulary $\left\{\mathbf{s}_m\right\}_{m}^{M}$ are also called sources. Since we are concerned with image decomposition/segmentation problem **X**, **A** and **S** are assumed to be nonnegative. Unsupervised (a.k.a. blind) decomposition assumes that only multichannel image matrix **X** is available to decomposition algorithm. Such problem is also known as blind source separation (BSS)[14]. The BSS problem (1) is ill-posed since matrix factorization implied by (1) suffers from indeterminacies: $\mathbf{X}=\mathbf{AS}=\mathbf{AB}^{-1}\mathbf{BS}$ for some $M\times M$ square invertible matrix **B**. Thus, the unsupervised image decomposition/segmentation problem has an infinite number of solutions. Meaningful solutions are characterized by the permutation and scaling indeterminacies in which case $\mathbf{B}=\mathbf{P}\mathbf{\Lambda}$, where **P** represents permutation and $\mathbf{\Lambda}$ represents diagonal scaling matrix. Such decomposition is said to be essentially unique[15]. However, constraints are necessary to be imposed on **A** and/or **S** in (1) to obtain solution of BSS problem that is unique up to permutation and scaling indeterminacies only[15]. In addition to nonnegativity constraint of **A** and **S** sparseness constraint imposed on **S** ensures, in principle*, decomposition (1) that is essentially unique. That leads to sparseness constrained nonnegative matrix factorization (sNMF)[8,16,17,18]. Sparseness constraint implies that only "small" amount of entries of matrix **S** is nonzero. In particular, when **X** in (1) represents RGB color microscopic image of the pathological specimen it is experimentally justified to assume that at each pixel only one source (histological structure) is present. Thus, rows of matrix **S** are orthogonal, i.e.

* In fact, as it is seen in supplementary note 2, mutual coherence of **A** determines amount of sparseness of **S** necessary to obtain essentially unique decomposition (1).

$s_{m_1 p} s_{m_2 p} = \delta(m_1 - m_2) \times c(p)$ where $\delta(m_1 - m_2)$ stands for Kronecker delta and c(p) is constant that depends on pixel location. Since we are interested to solve image segmentation problem we further assume $c(p) = 1$, $\forall p=1,...,P$. Thus, it is assumed that sources are orthogonal and binary:

$$s_{m_1 p} s_{m_2 p} = \delta(m_1 - m_2) \;\; \forall p=1,...,P \text{ and } \forall m_1, m_2 = 1,...,M \qquad (2).$$

SUPPLEMENTARY MATERIAL 2: MUTUAL COHERENCE OF BASIS (MIXING) MATRIX, SPECTRAL DIVERSITY AND UNIQUENESS OF MULTICHANNEL IMAGE DECOMPOSITION

To understand difficulties associated with decomposition of RGB color microscopic image of unstained specimen it is necessary to study conditions that guarantee solution of the linear system (1) that is unique as well as stable and/or robust to the presence of noise. To this end, we write (1) at some arbitrary pixel position:

$$\mathbf{x}_p = \mathbf{A}\mathbf{s}_p \qquad p = 1,...,P \qquad (3)$$

where $\mathbf{x}_p \in \mathbb{R}_{0+}^{N \times 1}$ and $\mathbf{s}_p \in \mathbb{R}_{0+}^{M \times 1}$. At the moment we shall assume that basis matrix $\mathbf{A}$ is known. Important implication of (2) is that (3) can be written as:

$$\mathbf{x}_p = \mathbf{a}_{m(p)} \qquad (4)$$

where $m(p) \in \{1,...,M\}$ denotes index of the source that is present at pixel p. In case of RGB image, N stands for number of color channels and equals N=3. M stands for number of *spectrally distinct* histological structures present in the specimen and for most scenarios it satisfies $M \in \{4, 5, 6\}$. Thus, we have $M>N$. Hence, to obtain unique solution of (3) $\mathbf{s}_p$ has to be sparse. That is [19,20,21]:

$$\left\| \mathbf{s}_p \right\|_0 < \frac{1}{2}\left(1 + \frac{1}{\mu(\mathbf{A})}\right) \qquad (5)$$

where $\left\| \mathbf{s}_p \right\|_0 = \#\{m : s_{mp} \neq 0\}_{m=1}^{M}$ counts number of non-zero entries of $\mathbf{s}_p$ and $0 \leq \mu(\mathbf{A}) \leq 1$ stands for mutual coherence of $\mathbf{A}$:

$$\mu(\mathbf{A}) = \max_{i,j} \{c_{ij}\}_{i=1, j=i+1}^{M-1, M} \qquad (6)$$

$$c_{ij} = \frac{\langle \mathbf{a}_i, \mathbf{a}_j \rangle}{\left\| \mathbf{a}_i \right\| \left\| \mathbf{a}_j \right\|}$$

where $\langle \mathbf{a}_i, \mathbf{a}_j \rangle$ stands for inner product between $\mathbf{a}_i$ and $\mathbf{a}_j$ and $\|\mathbf{a}_i\|$ stands for ℓ_2-norm of $\mathbf{a}_i$. Mutual coherence $\mu(\mathbf{A})$ measures worst case similarity between basis vectors $\{\mathbf{a}_m\}_{m=1}^M$. It determines, through (5), the amount of sparseness of $\mathbf{s}_p$ necessary to obtain unique solution of (3). Within the context of the present paper the intention is to emphasize that values of $\mu(\mathbf{A})$ "very close" to 1 drastically reduce possibility to obtain solution that is meaningful. Such situation indeed occurs when in (1) $\mathbf{X}$ represents RGB color microscopic image of unstained specimen. In this case basis vectors $\{\mathbf{a}_m\}_{m=1}^M$ stand for spectral profiles of the histological structures present in the specimen. When specimen is not stained these spectral profiles become highly correlated, i.e. corresponding histological structures are very hard to distinguish. That is, the visual cues inferred from segmented and digitally stained image of the unstained specimen cannot distinguish healthy and disease tissue. As an example we consider RGB image of unstained section of a specimen of human hepatocellular carcinoma and that is shown in figure 2a. By virtue of (4) and by knowing spatial locations of particular histological structures (they can be inferred from H&E stained image shown in figure 2e) we have estimated their spectral profiles, i.e. the basis matrix $\mathbf{A}$ in (1)/(3). Afterwards, we have estimated mutual coherence as $\mu(\mathbf{A})$=0.9999 and the average correlation between all the basis vectors as 0.9998.[†] For $\mu(\mathbf{A})$=0.9999 uniqueness condition (5) is formally satisfied for orthogonal sources stated by condition (2). In such case only one histological structure is present at each particular pixel, that is $\|\mathbf{s}_p\|_0 = 1$. The problems, however, arise when model (3), i.e. (1), holds only approximately. That occurs due to presence of additive noise or modeling errors. In such scenario $\mathbf{s}_p$ can be estimated by means of algorithms such as basis pursuit denoising (BPDN)[21,22]. Under assumption that model (3) holds up to the error tolerance $\delta>0$: $\|\mathbf{x}_p - \mathbf{A}\mathbf{s}_p\|_2 \leq \delta$ the BPDN algorithm tries to solve optimization problem:

$$\min_{\mathbf{s}_p} \frac{1}{2}\|\mathbf{x}_p - \mathbf{A}\mathbf{s}_p\|_2^2 + \gamma\|\mathbf{s}_p\|_1 \tag{7}$$

for some regularization constant γ. In such scenario good algorithm should yield estimate of $\mathbf{s}_p$, we denote it by $\mathbf{s}_p^\delta$, that contains at most non-zero entries as estimate of the $\mathbf{s}_p$ by the same algorithm from the exact model (3)[23]. In case of BPDN algorithm solution will be stable within the tolerance:

$$\|\mathbf{s}_p^\delta - \mathbf{s}_p\|_2^2 \leq \frac{4\delta^2}{1+\mu(\mathbf{A})\left(4\|\mathbf{s}_p\|_0 - 1\right)}$$

provided that sparseness of $\mathbf{s}_p$ satisfies[24,21]:

---

† By using the same procedure for image of unstained specimen of human liver with metastsis of colon cancer shown in figure 3a we have estimated $\mu(\mathbf{A})$=0.9997 and the average correlation between spectral profiles as 0.9993. Likewise, for image of unstained specimen of human liver with metastasis of gastric cancer shown in figure 4a we have estimated $\mu(\mathbf{A})$=0.9999 and the average correlation between spectral profiles as 0.9988.

$$\left\|\mathbf{s}_p\right\|_0 < \frac{1}{4}\left(1 + \frac{1}{\mu(\mathbf{A})}\right). \tag{8}$$

Thus, different amounts of mutual coherence $\mu(\mathbf{A})$ are required to satisfy uniqueness condition (5) and stability condition (8). In particular, for the pre-specified amount of sparseness $\left\|\mathbf{s}_p\right\|_0$ smaller value of $\mu(\mathbf{A})$ is required to satisfy (8) than to satisfy (5). We can further introduce the measurement noise in (3) and that yields: $\mathbf{x}_p=\mathbf{A}\mathbf{s}_p + \mathbf{w}_p$. Assuming that $\mathbf{w}_p$ is zero-mean white Gaussian with covariance $E\left\{\mathbf{w}_p\mathbf{w}_p^T\right\} = \sigma^2\mathbf{I}_{N\times N}$ estimation $\mathbf{s}_p^{BP}$ of BPDN (7) is unique with probability exceeding

$$\left(1-\left(M-\left\|\mathbf{s}_p\right\|_0\right)\exp\left(-\frac{\gamma^2}{8\sigma^2}\right)\right)\left(1-e^{-\left\|\mathbf{s}_p\right\|_0/7}\right)$$

when sparseness of $\mathbf{s}_p$ satisfies[25]:

$$\left\|\mathbf{s}_p\right\|_0 < \frac{1}{3\mu(\mathbf{A})}. \tag{9}$$

Comparatively to (8) and (5) even smaller value of $\mu(\mathbf{A})$ is required to satisfy (9) for a given $\left\|\mathbf{s}_p\right\|_0$. To this end, supplementary figure 7 shows amount of sparseness $\left\|\mathbf{s}_p\right\|_0$ as a function of mutual coherence $\mu(\mathbf{A})$ for conditions (5), (8) and (9). Since within the context of the present paper we have assumed that histological structures present in the image do not overlap, see (2), it follows $\left\|\mathbf{s}_p\right\|_0 = 1$. Thus, while condition (5) is satisfied even when $\mu(\mathbf{A})\approx 1$ it is seen that approximately $\mu(\mathbf{A})<0.33$ is required to satisfy (8) and (9). Even though these performance guarantees can be qualified as too pessimistic it is clear that possibly essentially unique decomposition of RGB image of unstained specimen into non-overlapping histological structures requires representation such as (1) but with basis matrix that is less coherent than **A**. We can further support this statement by the following argument. Under constraint $\left\|\mathbf{s}_p\right\|_0 = 1$ and assuming that **A** is estimated, the nonnegative orthogonal matching pursuit (NOMP) algorithm[26,8] can be used to solve (2):

$$\begin{aligned}
&\mathbf{d} = \mathbf{A}^{\mathrm{T}}\mathbf{x}_p \\
&m = \underbrace{\arg\max}_{i=1,..,M}\left\{d_i\right\} \\
&\mathbf{s}_p = \mathbf{e}_m
\end{aligned} \tag{10}$$

where $\mathbf{e}_m \in \mathbb{R}_{0+}^M$ stands for unit vector that is part of standard basis in Euclidean space. Evidently, when $\left\{\mathbf{a}_m\right\}_{m=1}^M$ are highly correlated, that is $\mu(\mathbf{A})\approx 1$, coefficients $\left\{d_i\right\}_{i=1}^M$ will be very close to each other. Let us suppose that $\mathbf{x}_p$ corresponds with histological structure m represented by basis vector $\mathbf{a}_m$. Then, very small amount of noise will cause that $\mathbf{x}_p$ be

correlated more with some basis vector $\mathbf{a}_n$ instead with the basis vector $\mathbf{a}_m$. That will imply $d_n > d_m$. Thus, $\mathbf{x}_p$ will be assigned to wrong histological structure by the NOMP algorithm. Therefore, we propose mathematical transform of the RGB microscopic image $\mathbf{X}$ that under linear mixture model representation (1) and constraints (2) yields:

$$\psi(\mathbf{X}) = \mathbf{BS} \tag{11}$$

whereat $\psi(\mathbf{X}) \in \mathbb{R}_{0+}^{D\times P}$, $\mathbf{B} \in \mathbb{R}_{0+}^{D\times M}$ and $D \geq M$ such that:

$$\mu(\mathbf{B}) < \mu(\mathbf{A}) \tag{12}.$$

SUPPLEMENTARY MATERIAL 3: EMPTIRICAL KERNEL MAP-BASED NONLINEAR MAPPING OF MULTICHANNEL IMAGE

Nonlinear mapping $\psi(\mathbf{X})$ to be derived herein has in many aspects already been presented within the framework on blind separation of analytes from linear and nonlinear underdetermined mixtures of mass spectra[27,28]. The essential difference, from the current paper point of view, is that orthogonality and binary constraint (2) imposed on histological structures (sources) $\{\mathbf{s}_m\}_{m=1}^M$ is stronger than sparseness constraints imposed on mass spectra of analytes[27,28]. Because of that and in order to make the present paper self-contained we shall present herein full derivation of the mapping $\psi(\mathbf{X})$ as well as its linear mixture model representation (11). It is clear that mapping $\psi(\mathbf{X})$ in (11) has to be nonlinear. Otherwise, channels of image $\psi(\mathbf{X})$ would be redundant combinations of channels of image $\mathbf{X}$. We propose pixel (column)-wise nonlinear mapping of $\mathbf{X}$:

$$\left\{\mathbf{x}_p \in \mathbb{R}_{0+}^{N} \mapsto \phi(\mathbf{x}_p) \in \mathbb{R}_{0+}^{\bar{N}}\right\}_{p=1}^{P} \text{ such that } \bar{N} \gg N. \tag{13}$$

In order to simplify notation pixel index p will be dropped in the subsequent derivations. The mapping $\phi(\mathbf{x})$ has the following structure:

$$\phi(\mathbf{x}) = \left[\left\{c_{q_1\ldots q_N} x_1^{q_1} \ldots x_N^{q_N}\right\}_{q_1,\ldots,q_N=0}^{\bar{N}}\right]^T \text{ such that } \sum_{n=1}^{N} q_n \leq \bar{N}. \tag{14}$$

In (13) $\left\{c_{q_1\ldots q_N}\right\}$ stand for mapping dependent coefficients. By taking into account that

$\left\{x_n = \sum_{m=1}^{M} a_{nm} s_m\right\}_{n=1}^{N}$, (14) can be written as:

$$\phi(\mathbf{x}) = c_0\mathbf{e}_1 + \mathbf{G}\begin{bmatrix} 0 \\ \mathbf{s} \end{bmatrix} + \mathbf{G}_{HOT}\begin{bmatrix} 0 \\ \mathbf{0}_{M\times 1} \\ \mathbf{s}_{HOT} \end{bmatrix} \tag{15}$$

where *HOT* stands for higher order (nonlinear) terms induced by mapping $\phi(\mathbf{x})$, $\mathbf{e}_1$ is a unit vector in standard Euclidean basis in $\mathbb{R}^{\bar{N}}$, $\mathbf{0}_{M\times 1}$ is column vector with zero entries and $\mathbf{s}_{HOT}$ is $\bar{N}-M-1$ column vector comprised of $\left\{s_1^{q_1}\times..\times s_M^{q_M}\right\}_{q_1,\dots,q_M=2}^{\bar{N}}$ and $\sum_{m=1}^{M} q_m \leq \bar{N}$. $\mathbf{G}$ and $\mathbf{G}_{HOT}$ in (15) are matrices of appropriated dimensions and are functions of the basis matrix $\mathbf{A}$ in (1) and mapping coefficients $\left\{c_{q_1\cdots q_N}\right\}$ in (14). Due to constraint (2) $\mathbf{s}_{HOT}$ in (15) simplifies to:

$$\mathbf{s}_{HOT} = \mathbf{s}. \tag{16}$$

Thus, pixel-wise nonlinear mapping $\phi(\mathbf{x})$ yields:

$$\phi(\mathbf{x}) = \begin{bmatrix} \mathbf{c} \\ \bar{\mathbf{G}} \end{bmatrix}\mathbf{s} \tag{17}$$

where $\mathbf{c} = \left[\underbrace{c_0 \dots c_0}_{\times M}\right]$ and $\bar{\mathbf{G}} \in \mathbb{R}_{0+}^{\bar{N}\times M}$ combines matrices $\mathbf{G}$ and $\mathbf{G}_{HOT}$ from (15) under constraint (16). We can generalize (17) to matrix level:

$$\phi(\mathbf{X}) = \begin{bmatrix} \mathbf{c} \\ \bar{\mathbf{G}} \end{bmatrix}\mathbf{S} \tag{18}$$

where $\phi(\mathbf{X}) \in \mathbb{R}_{0+}^{\bar{N}\times P}$. Hence, underdetermined blind source separation problem implied by (1) is substituted by new problem (18). Important difference is that number of (non-physical) channels $\bar{N}$ in (18) is greater than number of (physical) channels N in (1). Thus, by selection of mapping order $\bar{N}$ and type of mapping $\phi(\mathbf{x})$, which determines mapping coefficients $\left\{c_{q_1\cdots q_N}\right\}$, it should be possible to obtain basis matrix in (18) with mutual coherence that is smaller than $\mu(\mathbf{A})$ in (1). However, the difficulty with factorization of problem implied by (18) is that $\bar{N}$ can be very large or even infinite, in which case factorization becomes computationally intractable. To alleviate this difficulty a special type of nonlinear mapping $\phi(\mathbf{x})$ is sought for. To this end we reproduce some definitions and theorems[29].

Theorem 1. (Mercer's theorem)[30,29]. Consider the compact self-adjoint integral operator on $L^2(\Omega)$

$$\left(L_K f\right)(\mathbf{x}) = \int_\Omega K(\mathbf{x},\mathbf{y}) f(\mathbf{y})\,\mathbf{dy},$$

satisfying conditions:

C1: $\iint_{\Omega\times\Omega} \left|K(\mathbf{x},\mathbf{y})\right|^2 \mathbf{dxdy} < \infty$,

C2: $K(\mathbf{x},\mathbf{y})=K(\mathbf{y},\mathbf{x})$,

C3: $\Omega = \underbrace{[a,b]\times\ldots\times[a,b]}_{N\,times}$,

C4: $K(\mathbf{x},\mathbf{y})$ is continuous in $\mathbf{x}$ and $\mathbf{y}$.

If the eigenvalues $\{\lambda_n\}_{n\in\mathbb{N}}$ corresponding to the eigenfunctions $\{\phi_n(\mathbf{x})\}_{n\in\mathbb{N}}$ of operator L_K satisfy condition:

C5: $\lambda_n>0 \quad n\in\mathbb{N}$

then the kernel expansion

$$K(\mathbf{x},\mathbf{y}) = \sum_{n\in\mathbb{N}} \lambda_n \phi_n(\mathbf{x}) \phi_n(\mathbf{y}) \tag{19}$$

is uniformly convergent. □

Without loss of generality we further assume that in **C3:** a=0 and b=1.

Theorem 2. (Reproducing kernel Hilbert space, The Moore-Aronszajn theorem)[31,29]. There exists a uniquely determined Hilbert space H_K of functions on Ω satisfying the following conditions:

C1: $K(\circ,\mathbf{x}) \in H_K, \forall \mathbf{x}\in\Omega;$

C2: $f(\mathbf{x}) = \left\langle f, K(\circ,\mathbf{x})\right\rangle_{H_K}, \forall f\in H_K.$

where $\langle\circ,\circ\rangle$ denotes the inner product associated with H_K. □

Definition 1. Replacing $f(\mathbf{x})$ in **C2** in Theorem 2 by $K(\circ,\mathbf{x})$, it follows $K(\mathbf{y},\mathbf{x})=\langle K(\circ,\mathbf{y}),K(\circ,\mathbf{x})\rangle_{H_K}$. By selecting the nonlinear map as $\phi(\mathbf{x})=K(\circ,\mathbf{x})$ it follows:

$$K(\mathbf{y},\mathbf{x})=\langle \phi(\mathbf{y}),\phi(\mathbf{x})\rangle_{H_K} \tag{20}$$

Eq. (20) is known as *kernel trick*. The nonlinear mapping $\phi(\mathbf{x})$ is called explicit feature map (EFM). □

Kernel expansion (19) implied by Mercer's theorem can be interpreted on the following way[29]:

$$\begin{aligned}K(\mathbf{x},\mathbf{y}) &= \sum_{n\in\mathbb{N}}\sqrt{\lambda_n}\phi_n(\mathbf{x})\sqrt{\lambda_n}\phi_n(\mathbf{y}) \\ &=\left\langle \left\{\sqrt{\lambda_n}\phi_n(\mathbf{x})\right\}_{n\in\mathbb{N}},\left\{\sqrt{\lambda_n}\phi_n(\mathbf{y})\right\}_{n\in\mathbb{N}}\right\rangle_{\ell^2} \\ &=\langle \phi(\mathbf{x}),\phi(\mathbf{y})\rangle_{\ell^2}\end{aligned} \tag{21}$$

where ℓ^2 denotes feature space. Thus, when $K(\mathbf{x},\mathbf{y})$ is Mercer's kernel EFM $\phi(\mathbf{x})$ is composed of eigenvalues $\{\lambda_n\}_{n\in\mathbb{N}}$ and eigenfunctions $\{\phi_n(\mathbf{x})\}_{n\in\mathbb{N}}$ of $K(\mathbf{x},\mathbf{y})$, i.e. $\phi(\mathbf{x})=\left\{\sqrt{\lambda_n}\phi_n(\mathbf{x})\right\}_{n\in\mathbb{N}}$. The central observation is that inner product of two feature vectors in the infinite-dimensional space is given by the evaluation of the kernel as a function and that is known as *kernel trick*[32]. Normally, one does not need to design particular eigenvalues $\{\lambda_n\}_{n\in\mathbb{N}}$ and eigenfunctions $\{\phi_n(\mathbf{x})\}_{n\in\mathbb{N}}$ but chooses simple kernel function $K(\mathbf{x},\mathbf{y})$ that satisfies conditions of Mercer's theorem. One such example is the Gaussian kernel:

$$K(\mathbf{x},\mathbf{y})=\exp\left(-\|\mathbf{x}-\mathbf{y}\|_2^2/\sigma^2\right) \tag{22}$$

where σ^2 denotes kernel bandwidth or variance. Within the context of the present paper importance of the *kernel trick* is to transform factorization problem implied by (18) to the new one that is computationally tractable even when $\bar{N}\rightarrow\infty$. To this end, it is necessary to project EFM $\phi(\mathbf{x})$ on a subspace in RKHS H_K that is spanned by a basis $\left\{\phi(\mathbf{v}_d)\in\mathbb{R}^{\bar{N}}\right\}_{d=1}^{D}$:

$$\psi(\mathbf{x})=\left[\phi(\mathbf{v}_1)\ ...\ \phi(\mathbf{v}_D)\right]^T\phi(\mathbf{x})=\begin{bmatrix}K(\mathbf{x},\mathbf{v}_1)\\ ....\\ K(\mathbf{x},\mathbf{v}_D)\end{bmatrix} \tag{23}$$

The mapping (23) $\psi:\mathbb{R}^N \rightarrow \mathbb{R}^D$ is known as empirical kernel map (EKM) with respect to basis $\left\{\mathbf{v}_d \in \mathbb{R}^N\right\}_{d=1}^D$: $\mathbf{x} \mapsto K(\circ,\mathbf{x})\Big|_{\{\mathbf{v}_d\}_{d=1}^D} = \left[K(\mathbf{v}_1,\mathbf{x}),...,K(\mathbf{v}_D,\mathbf{x})\right]^T$, see also definition 2.15 in ref. 32. When (18) is substituted in (23) we obtain:

$$\psi(\mathbf{x}) = \left[\phi(\mathbf{v}_1)\ ...\ \phi(\mathbf{v}_D)\right]^T \phi(\mathbf{x}) = \left[\phi(\mathbf{v}_1)\ ...\ \phi(\mathbf{v}_D)\right]^T \begin{bmatrix}\mathbf{c}\\ \overline{\mathbf{G}}\end{bmatrix}\mathbf{s} \tag{24}$$
$$= \mathbf{Bs}$$

We can write (24) in the matrix version as:

$$\psi(\mathbf{X}) = \mathbf{BS} \tag{25}$$

where $\psi(\mathbf{X}) \in \mathbb{R}_{0+}^{D\times P}$, $\mathbf{B} \in \mathbb{R}_{0+}^{D\times M}$ and $\mathbf{S} \in \mathbb{R}_{0+}^{M\times P}$. Linear mixture model based representation of $\psi(\mathbf{X})$ in (25) matches the one postulated in (11).

Projection implied by (23) demands a basis $\mathbf{V} = \left\{\mathbf{v}_d \in \mathbb{R}_{0+}^N\right\}_{d=1}^D$ in the vector space spanned by the empirical data set $\left\{\mathbf{x}_p\right\}_{p=1}^P$ such that

$$span\left\{\mathbf{v}_d\right\}_{d=1}^D \approx span\left\{\mathbf{x}_p\right\}_{p=1}^P \tag{26}$$

where *span* denotes a vector space spanned by particular set of vectors. Thus, it is expected that basis vectors span the same vector space as the one spanned by empirical set of patterns. When (26) holds it is expected that the following will also hold:

$$span\left\{\phi(\mathbf{v}_d)\right\}_{d=1}^D \approx span\left\{\phi(\mathbf{x}_p)\right\}_{p=1}^P \tag{27}$$

The basis **V** can be constructed on several ways. One way is by using data clustering whereas cluster centers represent basis vectors. In this regard, results shown in figures 1 to 4 as well as supplementary figures 2a, 5e and 6f are obtained by using basis selected by *k-means* clustering algorithm[34] implemented by a MATLAB function kmeans.

When it comes to the kernel function necessary to compute $\psi(\mathbf{x})$ in (23) we have used the Gaussian kernel (22) that, arguably, is most often used kernel in machine learning[33]. Thus, EKM $\psi(\mathbf{x})$ in (23)-(25) is parameterized by subspace dimension D and kernel variance σ^2, whereat kernel variance σ^2 has dominant importance. That is, it is shown in figures 1f and 2f that the value of $\mu(\mathbf{B})$ virtually depends on the value of variance σ^2 of the Gaussian kernel (22). However, only naive interpretation of these results suggests that by decreasing σ^2 towards zero we could obtain $\mu(\mathbf{B})$=0, i.e. **B** to be orthogonal. In practice, however, we map **X**

through (23). Thereby, $\left\{K\left(\mathbf{x},\mathbf{v}_d\right)\right\}_{d=1}^{D}$ measures similarity between pixel vector **x** and basis vectors $\left\{\mathbf{v}_d\right\}_{d=1}^{D}$. The basis vectors stand for cluster centers and represent in average pixels within the clusters, i.e. histological structures. They are in case of RGB image of unstained specimen highly correlated. Due to the presence of noise and variability with the specimen itself actual pixel **x** could be closer to some cluster center $\mathbf{v}_m$ than to cluster center $\mathbf{v}_n$ with which it actually corresponds. Thus, if σ^2 is too small $K\left(\mathbf{x},\mathbf{v}_m\right)$ will be close to 1 and $K\left(\mathbf{x},\mathbf{v}_n\right)$ close to 0 and that is wrong. Essentially, value of the kernel bandwidth σ^2 has to tradeoff between being large enough to provide robustness to noise and specimen variability and being small enough to decrease $\mu(\mathbf{B})$. That, indeed, is confirmed in supplementary figure 1. It shows color coded results of segmentation of synthetic image shown in figure 1a and obtained by means of EKM-NMU algorithm with D=20. Results are shown for combination of variance of Gaussian kernel σ^2 and highest SNR value in dB for which obtained result still differs from ground truth shown in supplementary figure 1a. The values of σ^2 and SNR that respectively correspond with supplementary figures 1b to 1c are (10^{-3}, 28 dB), (10^{-2}, 17 dB) and (10^{-1}, 13 dB). Thus, SNR values for which correct segmentation results are obtained are given respectively as 29 dB, 18 dB and 14 dB. Hence, by increasing variance of Gaussian kernel in EKM-based mapping noise robustness has to be increased significantly. Thereby, $\mu(\mathbf{B})$ was still small enough to enable accurate segmentation. Furthermore, we show in supplementary figure 2f images of color coded segmentation results obtained by EKM-NMF_L0 algorithm for σ^2=0.01 (left column) and σ^2=0.1 (right column). Thereby, dimension D of $\psi\left(\mathbf{X}\right)$ induced vector space is respectively from top to bottom: 150, 100, 50 and 25. While decomposition results in right column correspond qualitatively to ground truth image shown in figures 2b and 2e, decomposition results in left column are too noisy and qualitatively incorrect. To this end, results related to decompositions of images of unstained specimens shown in figures 2, 3 and 4 as well as supplementary figures 5 and 6 were obtained with D=50 and σ^2=0.1.

SUPPLEMENTARY MATERIAL 4: SPARSENESS AND NON-NEGATIVITY CONSTRAINED FACTORIZATION

After EKM-based nonlinear mapping of the RGB color microscopic image **X** (1), decomposition problem is reduced to non-negativity and sparseness constrained factorization of $\psi(\mathbf{X})$ in (25). That is, we have to estimate $\mathbf{B}\geq\mathbf{0}$ and of $\mathbf{S}\geq\mathbf{0}$ such that: $s_{mp}s_{np}=\delta(m-n)$, $\forall m,n=1,...,M$ and $\forall\ p=1,...,P$. To this end, solution of (25) can be approached on two principal ways known as sparse component analysis (SCA) and NMF. Even though results related to segmentation of image of unstained specimen, presented in figures 2 to 4 and supplementary figures 2 to 6, were obtained by means of sparseness constrained NMF of $\psi(\mathbf{X})$, we shall (to make this material self-contained) present brief overview of both approaches.

SUPPLEMENTARY MATERIAL 4.1: Sparse component analysis

SCA methods estimate basis matrix **B** first and then solve for encoding coefficients **S** by solving optimization problems such as NOMP in (10). However, as it is evident from (23)/(24), **B** as well as ψ(**X**), demand estimate of the basis $\mathbf{V} \in \mathbb{R}_{0+}^{N\times D}$ that, according to (26), spans approximately the same vector space as the empirical set of patterns **X**. When D=M, **V** may coincide with the true basis matrix **A** in (1). Within the context of the present paper **V** is estimated by clustering empirical set of patterns $\left\{\mathbf{x}_p\right\}_{p=1}^{P}$ into D clusters with cluster centers $\left\{\mathbf{v}_d\right\}_{d=1}^{D}$. For this purpose *k-means* clustering algorithm[34] implemented by a MATLAB function kmeans has been used. Since $\left\{\mathbf{v}_d\right\}_{d=1}^{D}$ spans basis in the input patterns space $\mathbb{R}_{0+}^{N}$ $\left\{\phi\left(\mathbf{v}_d\right)\right\}_{d=1}^{D}$ will span the basis in possibly infinitely dimensional image pattern space, i.e. (27) is expected to hold. Thus, (17) can be also written as:

$$\phi(\mathbf{x}) \approx \phi(\mathbf{V})\mathbf{s} \tag{28}$$

where $\phi(\mathbf{V}) := \left\{\phi\left(\mathbf{v}_d\right)\right\}_{d=1}^{D}$. It follows from (24)/(25) that:

$$\mathbf{B} = \phi(\mathbf{V})^{\mathrm{T}} \phi(\mathbf{V}) = \psi(\mathbf{V}) \tag{29}$$

i.e. that **B** is the EKM-based mapping of **V**. Hence, to estimate encoding coefficients **S** in (24) it suffices to solve the sparse coding problem in mapping induced RKHS. By taking into account that $\left\{\left\|\mathbf{s}_p\right\|_0 = 1\right\}_{p=1}^{P}$ the sparse coding problem can be solved for each pixel p=1,...,P by NOMP algorithm such as (10):

$$\begin{aligned} &\mathbf{c} = \mathbf{B}^T \psi\left(\mathbf{x}_p\right) \\ &m = \underbrace{\arg\max}_{d=1,..,D} \left\{c_d\right\} \\ &\mathbf{s}_p = \mathbf{e}_m \end{aligned} \tag{30}$$

where $\mathbf{e}_m \in \mathbb{R}_{0+}^{D}$ stands for unit vector that is part of standard basis in Euclidean space. Since μ(**B**)< μ(**A**) it is expected that in the presence of noise the NOMP-based solution (30) will coincide better with the true solution than the NOMP-based solution (10) will do.

SUPPLEMENTARY MATERIAL 4.2: Sparseness constrained NMF

NMF optimization problem (1)/(25) can be formalized as[35]:

$$\underbrace{\min}_{\mathbf{W},\mathbf{S}} \left\| \mathbf{Y} - \mathbf{WS} \right\|_F \qquad (31)$$

subject to

$$\mathbf{W} \geq \mathbf{0}, \quad \mathbf{S} \geq \mathbf{0}$$

where F stands for Froebenius norm and $\geq$ denotes element-wise greater than or equal to operator. If (31) refers to (1) **Y=X** and **W**=**A**. If (31) refers to (25) **Y**=ψ(**X**) and **W**=**B**. $\left\| \mathbf{Y} - \mathbf{WS} \right\|_F$ is nondecreasing under update rules[35]:

$$\mathbf{S} \leftarrow \mathbf{S} \otimes \frac{\mathbf{W}^{\mathrm{T}}\mathbf{Y}}{\mathbf{W}^{\mathrm{T}}\mathbf{WS}}$$

$$\mathbf{W} \leftarrow \mathbf{W} \otimes \frac{\mathbf{Y}\mathbf{S}^{\mathrm{T}}}{\mathbf{WS}\mathbf{S}^{\mathrm{T}}} \qquad (32)$$

where $\otimes$ denotes element-wise multiplication and / denotes element-wise division. Obviously, for nonnegative initial values of **W** and **S** multiplicative update rules (32) ensure nonnegativity of **W** and **S** automatically. Since NMF allows additive combinations only its solution often yields parts based representation, that is rows of **S** and/or columns of **W** correspond with different parts present in **X**. Also, quite often parts-based representation is sparse but that is obtained as a by-product of (31). To decompose color microscopic image of unstained specimen into non-overlapping histological structures that coincide with the rows of **S** it is necessary to strictly enforce sparseness of $\left\{ \mathbf{s}_m \right\}_m^M$ during optimization procedure.

SUPPLEMENTARY MATERIAL 4.2.1: Sparse NMF with ℓ_0-constraints

Natural measure of sparseness is ℓ_0-norm that counts number of non-zero entries of some vector/matrix. To this end optimization problem (31) can be extended to[8]:

$$\underbrace{\min}_{\mathbf{W},\mathbf{S}} \left\| \mathbf{Y} - \mathbf{WS} \right\|_F$$

subject to:

$$\mathbf{W} \geq \mathbf{0}, \quad \mathbf{S} \geq \mathbf{0}$$

$$\left\| \mathbf{s}_p \right\|_0 \leq L \quad \forall p = 1, \ldots, P \qquad (33)$$

where L stands for maximal number of non-zero entries. Within the context of the present paper it applies L=1. Optimization problem (33) can be described with the following procedure[8]:

Algorithm NMF_L0.
Step 1. Initialize **W** randomly
Step 2. for i=1: numIter **do**
Step 3. Nonnegative sparse coding: Encode sparsely data **Y** using fixed matrix **W**. This yields sparse matrix **S**.
Step 4. Update of basis matrix W. Use update rule for **W** in (32).

end for

Nonnegative sparse coding step can be accomplished by NOMP algorithm with L=1 such as in (10) and/or (30). That is also called sparse nonnegative least square (sNNLS)[8]. Alternatively, reverse sNNLS[8] can be used to solve nonnegative sparse coding step in Algorithm NMF_L0. The rsNNLS is summarized in Algorithm rsNNLS. Initial non-sparse estimate of $\mathbf{s}_p$ is obtained by means of NNLS such as active-set NNLS algorithm[36]. Since $\mathbf{s}_p$ is non-sparse $\left\|\mathbf{s}_p\right\|_0 > 1$ the smallest coefficient is set to 0. Its index is moved from in-active set $\mathcal{I}$ to active set $\mathcal{Z}$. Data vector $\mathbf{y}_p$ is approximated in NNLS sense by the remaining basis vectors in $\mathcal{I}$. All results generated by means of the NMF_L0 algorithm were obtained by using reverse sNNLS in the nonnegative sparse coding step.

Algorithm rsNNLS.
Step 1. $\mathbf{s}_p$=NNLS($\mathbf{y}_p$, **W**)
Step 2. $\mathcal{Z}=\{i|s_{ip}=0\}$, $\mathcal{I}=\{i|s_{ip}>0\}$
Step 3. while $\left\|\mathbf{s}_p\right\|_0 > 1$ **do**
Step 4. $j = \arg\min_{i \in I} s_{ip}$
Step 5. s_{jp}=0.
Step 6. $\mathcal{Z} \leftarrow \mathcal{Z} \cup \{j\}$
Step 7. $\mathcal{I} \leftarrow \mathcal{I} \setminus \{j\}$
Step 8. $\mathbf{s}_{p(\mathcal{I})}$=NNLS($\mathbf{y}_p$, $\mathbf{W}_{(\mathcal{I})}$)
end while

When NMF_L0 algorithm outlined above has been applied to problem (25) we have named it the EKM-NMF_L0. The importance of applying the NMF_L0 algorithm in the RKHS space (25) instead in input space (1) is explained by the NOMP (10)/(30) that is recognized as sNNLS with L=1. It is also part of the reverse sNNLS algorithm because it is a part of the NNLS algorithm. In both cases index of nonzero coefficient in the solution vector $\mathbf{s}_p$ corresponds with the index of the maximal element of $\mathbf{W}^T\mathbf{y}_p$. Error in this crucial step is less probable when $\mu(\mathbf{W})$ is lower. Thus, since $\mu(\mathbf{B}) < \mu(\mathbf{A})$ NMF_L0 based solution of (25) is expected to be more accurate than NMF_L0 based solution of (1). In the extreme case when $\mu(\mathbf{A}) \approx 1$ NMF_L0, or any other method that factorizes (1) directly, will yield solution **S** that corresponds with ground truth very poorly. The Matlab implementation of the NMF_L0 algorithm is available for free download[37].

SUPPLEMENTARY MATERIAL 4.2.2: Sparse NMF with underapproximation constraints

The nonnegative matrix underapproximation (NMU) algorithm[18] has been applied to (1), respectively (25), in decomposition of synthetic image shown in figure 1a with results shown in figure 1d, respectively figures 1c. Furthermore, robustness of the EKM-NMU with respect to the presence of noise has been tested in supplementary figure 1. The reason for using the NMU algorithm instead of the NMF_L0 algorithm, that was used to decompose images of unstained specimens of human and mouse liver, was to demonstrate that separation of spectrally highly similar objects is primarily consequence of EKM-based mapping and not of sparseness constrained NMF algorithm. Unlike other sparseness constrained NMF algorithms[17,38], the NMU algorithm does not require explicit sparseness related regularization term. Thus, tuning of regularization constant, that is seen as limitation of many NMF algorithms[39], is not required. In relation to the NMF problem (31) the NMU algorithm imposes an additional underapproximation constraint on **W** and **S**: **WS≤Y**. That this, the NMU optimization problem is:

$$\underbrace{\min}_{\mathbf{W},\mathbf{S}} \left\| \mathbf{Y} - \mathbf{W}\mathbf{S} \right\|_F$$

subject to:

$$\mathbf{W} \geq \mathbf{0}, \;\; \mathbf{S} \geq \mathbf{0}, \;\; \mathbf{WS} \leq \mathbf{0} \qquad (34)$$

The NMU method performs factorization of (31) in a recursive manner extracting one component at a time. After identifying optimal rank-one solution $(\mathbf{w}_1, \mathbf{s}_1)$, the rank-one factorization is performed on the residue matrix $\mathbf{Y} \leftarrow \mathbf{Y} - \mathbf{w}_1\mathbf{s}_1$. Herein, $\mathbf{w}_1$ denotes column vector and $\mathbf{s}_1$ denotes row vector. Underapproximation constraint preserves non-negativity of **Y**. This constraint yields localized parts-based decomposition, where different basis vectors describe disjoint parts of the input data **Y**. It has been proven in theorem 1 in ref. 18 that, due to underapproximation constraint, sparseness (number of non-zero entries) of **W** and **S** is less than sparseness of **Y**. That enables sparseness constrained factorization without regularization constant. The Matlab implementation of the NMU algorithm is available for free download[40]. For the sake of comprehensiveness we reproduce the main steps of the NMU algorithm herein. It finds approximate solution of the optimization problem (34) by minimizing Lagrangian:

$$L(\mathbf{W}, \mathbf{S}, \mathbf{\Lambda}) = \frac{1}{2} \left\| \mathbf{Y} - \mathbf{W}\mathbf{S} \right\|_F^2 + \sum_{n=1}^{N} \sum_{p=1}^{P} \Lambda_{np} \left(\mathbf{W}\mathbf{S} - \mathbf{Y} \right)_{np}$$

where $\mathbf{\Lambda}$ stands for matrix of Lagrange multipliers. **W** is iteratively updated column-wise through:

$$\mathbf{w}_k = \max\left(\mathbf{0}, \frac{\mathbf{c}_k - \sum_{m=1, m\neq k}^{M} \mathbf{w}_m d_{mk}}{d_{kk}}\right) \tag{35}$$

where $\mathbf{C}=(\mathbf{Y}-\Lambda)\mathbf{S}^{\mathrm{T}}$ and $\mathbf{D}=\mathbf{S}\mathbf{S}^{\mathrm{T}}$. $\mathbf{S}$ is iteratively updated row-wise through:

$$\mathbf{s}_k = \max\left(\mathbf{0}, \frac{\mathbf{f}_k - \sum_{m=1, m\neq k}^{M} g_{km} \mathbf{s}_m}{g_{kk}}\right) \tag{36}$$

where $\mathbf{F}=\mathbf{W}^{\mathrm{T}}(\mathbf{Y}-\Lambda)$ and $\mathbf{G}=\mathbf{W}^{\mathrm{T}}\mathbf{W}$. Matrix of Lagrange multipliers Λ is updated iteratively through:

$$\Lambda \leftarrow \max\left(\mathbf{0}, \Lambda - \frac{1}{l}(\mathbf{Y}-\mathbf{WS})\right) \tag{37}$$

where l denotes iteration index. The algorithm starts with the initial values $\Lambda=\mathbf{0}$, and $\mathbf{W}$ and $\mathbf{S}$ such that $\mathbf{WS}\leq\mathbf{Y}$. The number of sources M has also to be supported to the algorithm as an input information.

SUPPLEMENTARY MATERIAL 4.2.3: Binarization of EKM-NMF separated components

NMF based decomposition of (25), the EKM-NMF_L0 or EKM-NMU, yields analog values of coefficients in code matrix $\mathbf{S}$. Even though these values are quite close to binary ones, imposed by constraint (2), that has to be ensured by thresholding analog values. To this end, to comply with orthogonality and binary constraint (2) column- (pixel) wise mapping is performed: $\left\{\mathbf{s}_p \mapsto \overline{\mathbf{s}}_p\right\}_{p=1}^{P}$, such that:

$$\overline{s}_{mp} = \begin{cases} 1 & for\ \ m = \arg\max_i \left\{s_{ip}\right\}_{i=1}^{M} \\ 0 & otherwise. \end{cases} \tag{38}$$

REFERENCES

[1] The MathWorks® Website. http://www.mathworks.com/help/images/examples/color-based-segmentation-using-k-means-clustering.html; Chitade, A.Z. & Katiyar, S.K. *Int. J. Eng. Sci. Tech.* **2**, 5319-5325 (2010).

[2] Osher, S., and Fedkiw, R., *Level Set Methods and Dynamic Implicit Surfaces*, Cambridge University Press, New York, NY, 2003.

[3] Sandhu, R., Georgiu, T., and Tannebaum, A., "A New Distribution Metric for Image Segmentation," in *Proc. SPIE 6914*, Medical Imaging 2008: Image Processing, 691404 (11 March 2008); doi: 10.1117/12.769010.

[4] The Romeil Sandhu Website at File Exchange Matlab Central. http://www.mathworks.com/matlabcentral/fileexchange/authors/54548 [4 March 2015].

[5] Chan, T.F., Sandberg, B.Y., and Vese, L.A., "Active Contours without Edges for Vector-Valued Images," *J. Visual Comm. Image Rep.* **11**, 130-141 (2000).

[6] The Yue Wu Website at File Exchange Matlab Central. http://www.mathworks.com/matlabcentral/fileexchange/23445-chan-vese-active-contours-without-edges [22 September 2014].

[7] McCann, M.T., Mixon, D.G., Fickus, M.C., Castro, C.A., Ozolek, J.A., and Kovačević, J., "Images as Occlusions of Textures: A Framework for Segmentation," *Image Processing, IEEE Transaction on* **23** (5), 2033-2046 (2014).

[8] Peharz, R., and Pernkopf, F., "Sparse nonnegative matrix factorization with ℓ^0-constraints," *Neurocomputing* **80**, 38-46 (2012).

[9] Begelman, G., Zibulevsky, M., Rivlin, E., and Kolatt, T., "Blind Decomposition of Transmission Light Microscopic Hyperspectral Cube Using Sparse Representation," *Medical Imaging*, *IEEE Transactions on* **28** (8), 1317-1324 (2009).

[10] Kopriva, I. and Peršin, A., "Unsupervised decomposition of low-intensity low-dimensional multi-spectral fluorescent images for tumour demarcation," *Medical Image Analysis* **13**, 507-518 (2009).

[11] Kopriva, I. and Cichocki, A., "Blind decomposition of low-dimensional multi-spectral image by sparse component analysis," *Journal of Chemometrics* **23** (11) 590-597 (2009).

[12] Nakai, T., Muraki, S., Bagarinao, E., Miki, Y., Takehara, Y., Matsuo, K., Kato, C., Sakahara, H., and Isoda, H., "Application of independent component analysis to magnetic resonance imaging for enhancing the contrast of gray and white matter," *Neuroimage* **21**, 251-260 (2004).

[13] Hu, X., Shimizu, A., Kobatake, H., and Nawano, S., "Independent Component Analysis of Four-Phase Abdominal CT Images," *Lecture Notes on Computer Science* **3217**, 916-924 (2004).

[14] Comon, P. and Jutten, H. (eds.). *Handbook on Blind Source Separation - Independent Component Analysis and Applications*, Academic Press (2010).

[15] Moreau, E. and Comon, P. *Contrasts*, Chapter 3 in *Handbook on Blind Source Separation - Independent Component Analysis and Applications*, Comon, P. and Jutten C. (Eds.), Academic Press (2010), pp. 65-105.

[16] Sajda, P., Du, S., Brown, D.T.R., Stoyanova, R., Shungu, D.C., Mao, X., and Parra, L.C., "Nonnegative Matrix Factorization for Rapid Recovery of Constituent Spectra in Magnetic Resonance Chemical Shift Imaging of the Brain," *Medical Imaging*, *IEEE Transactions on* **23** (12), 1453-1465 (2004).

[17] Hoyer, P., "Non-negative Matrix Factorization with Sparseness Constraints," *Journal of Machine Learning Research* **5**, 1457-1469 (2004).

[18] Gillis, N., and Glineur, F., "Using underapproximations for sparse nonnegative matrix factorization," *Pattern Recognition* **43**, 1676-1687 (2010).

[19] Gribonval, R., and Nielsen, M., "Sparse Representations in Unions of Bases," *Information Theory*, *IEEE Transactions on* **49** (12), 3320-3325 (2003).

[20] Tropp, J.A., "Greed is good: Algorithmic results for sparse approximation," *Information Theory*, *IEEE Transactions on* **50**, 2231-2242 (2004).

[21] Bruckstein, A.M., Donoho, D.L., and Elad, M., "From Sparse Solutions of Systems of Equations to Sparse Modeling of Signals and Images," *SIAM Review* **51** (1), 34-81 (2009).

[22] Tibshirani, R., "Regression shrinkage and selection via the Lasso," *J. Roy. Statist. Soc. B* **58** (1), 267-288 (1996).

[23] Chen, S.S., Donoho, D.L., and Saunders, M.A., "Atomic decomposition by basis pursuit," *SIAM J. Sci. Comput.* **20**, 33-61 (1998).

[24] Donoho, D.L., Elad, M., and Temlyakov, V., "Stable recovery of sparse overcomplete representations in the presence of noise," *Information Theory*, *IEEE Transactions on* **52**, 6–18 (2006).

[25]Ben-Haim, Z., Eldar, Y.C., and Elad, M., "Coherence-Based Performance Guarantees for Estimating a Sparse Vector Under Random Noise," *Signal Processing, IEEE Transactions on* **58** (10), 5030-5043 (2010).

[26] Bruckstein, A.M., Elad, M., and Zibulevsky, M., "On the uniqueness of nonnegative sparse solutions to underdetermined systems of equations," *Information Theory, IEEE Transactions on* **54**, 4813–4820, 2008.

[27] Kopriva, I., Jerić, I., and Brkljačić, L., "Nonlinear Mixture-wise Expansion Approach to Underdetermined Blind Separation of Nonnegative Dependent Sources," *J. of Chemometrics* **27** (8), 189-197 (2013).

[28] Kopriva, I., Jerić, I., Filipović, M., and Brkljačić, L., "Empirical Kernel Map Approach to Nonlinear Underdetermined Blind Separation of Sparse Nonnegative Dependent Sources: Pure Components Extraction from Nonlinear Mixtures Mass Spectra," *J. of Chemometrics* , **28** (9), 704-715 (2014).

[29] Kennedy, R.A., and Sadeghi, P., *Hilbert Space Methods in Signal Processing*, Cambridge University Press, 2013, pp. 321-351.

[30] Mercer, J.,"Functions of positive and negative type, and their connection with the theory of integral equations," *Philosoph. Trans. Roy. Soc. Lond*. **209**, 415-446 (1909).

[31] Aronszajn, N., "The theory of reproducing kernels," *Trans. of the Amer. Math. Soc*. **68**, 337-404 (1950).

[32] Aizerman, A., Braverman, E.M., and Rozoner, L.I., "Theoretical foundations of potential function method in pattern recognition learning," *Automatic and Remote Control* **25**, 821-837 (1964).

[33] Schölkopf, B., and Smola, A., *Learning with kernels*, MIT Press, 2002, pp. 42-45.

[34] Bishop, C.M., *Pattern Recognition and Machine Learning*, Springer, 2006, pp. 424-430.

[35] Lee, D.D., and Seung, H.S., "Learning the parts of objects by non-negative matrix factorization," *Nature* **401**(6755), 788-791 (1999).

[36] Lawson, C., and Hanson, R., *Solving Least Squares Problems*, Prentice-Hall, 1974.

[37] The Robert Peharz Website. https://www.spsc.tugraz.at/tools/nmf-l0-sparseness-constraints [4 March 2015].

[38] Cichocki, A., Zdunek, R., and Amari, S.I., "Hierarchical ALS. Algorithms for nonnegative matrix factorization and 3D tensor factorization," *LNCS* **4666**, 169–176 (2007).

[39] Yang, Z., Xiang, Y., Xie, S., Ding, S., and Rong, Y., "Nonnegative blind source separation by sparse component analysis based on determinant measure," *Neural Net. and Learn. Sys., IEEE Trans. on* **23** (10), 1601–1610 (2012).

[40] The Nicolas Gillis Website. https://sites.google.com/site/nicolasgillis/code [4 March 2015].
